# Recovery‐Oriented Conversations in Psychiatric Care: An Integrated Systematic Review

**DOI:** 10.1111/inm.70295

**Published:** 2026-06-22

**Authors:** Promise Ezinne Ekezie, Alexander Rozental, Git‐Marie Ejneborn‐Looi, Ursula Werneke, Sebastian Gabrielsson

**Affiliations:** ^1^ Department of Health, Education and Technology Luleå University of Technology Luleå Sweden; ^2^ Department of Clinical Neuroscience Karolinska Institute Stockholm Sweden; ^3^ Sunderby Research Unit, Department of Clinical Science, Psychiatry Umeå University Umeå Sweden

**Keywords:** communication, mental health recovery, mental health service, patient, psychiatric nursing

## Abstract

In psychiatric settings, recovery‐oriented care follows a relational, person‐centred approach. In mental health nursing, such care extends beyond merely reducing symptoms to foregrounding hope, identity, meaningfulness, empowerment, and personal agency. Because an important aspect of putting that approach into practice is engaging in conversations that promote recovery, understanding the concept of recovery‐oriented conversations (ROCs) is essential. In this review, we aim to synthesise existing research on ROCs and offer insights into their implementation, challenges, and impact on patients' recovery. An integrative review of studies (i.e., qualitative, quantitative, and mixed‐methods) published between 2015 and 2025 in PubMed, CINAHL, PsycINFO, Scopus, and Web of Science returned 5298 studies, 49 of which we included in a narrative synthesis using deductive coding from an inductive perspective. The findings suggest that ROCs are conceived as being well‐structured, context‐sensitive conversations that create space for expression and the co‐creation of recovery‐oriented pathways while probing personal and social realities. ROCs are effective when grounded in deep engagement, shared goal‐setting, and supportive environments. The practice, reflecting the connectedness, hope, identity, motivation and empowerment (CHIME) framework, is perceived as nurturing safety, promoting relational depth, and reaffirming personhood. Whereas barriers to implementation include limited time, staffing constraints, hierarchical cultures, and inconsistent leadership, facilitators include clear communication, culturally responsive practices, and organisational support. Altogether, ROCs allow mental health nurses to deliver care, strengthen therapeutic relationships, and foster shared decision‐making. Embedding them into successful practice, however, calls for more research, training, cultural sensitivity, and systemic alignment.

## 1 Introduction

Recovery‐oriented care is increasingly recognised as a pillar of contemporary mental health nursing. Instead of exclusively aiming to reduce symptoms, recovery‐oriented care extends into personal and social practices that encompass the rekindling of hope, identity, agency, and meaning in everyday life (Anthony 1993; Chester et al. 2016; Cleary et al. 2013; Davidson et al. 2005; Subandi et al. 2023). Such practices are manifested in the recovery‐focused principles modelled in the connectedness, hope, identity, meaning, and empowerment (CHIME) framework, which underscores how the relationships that people craft together, through narratives and self‐determination, shape their journeys towards personal recovery (Leamy et al. 2011). Such journeys, however, have been criticised for not adequately considering the broader social, political, and structural factors that may influence an individual's chances of recovery (Karadzhov 2023; Nakanishi et al. 2021). Some scholars have also argued that placing the burden of recovery solely on recovering individuals can be problematic, especially when they struggle to recover as expected, because it can leave them with feelings of failure and inadequacy (Karadzhov 2023; Melillo et al. 2025; Nakanishi et al. 2021). Mental health nurses are therefore urged to forge partnerships with patients that recognise them as the foremost experts on their own lived experiences and honour their perspectives, wishes, and objectives (Cleary et al. 2013; De Ruysscher et al. 2020).

At its core, therapeutic communication between nurses and patients should extend beyond a simple exchange of information and actively cultivate trust, validation, and genuine understanding. By listening, extending empathy, and reflecting on what patients share, nurses can help people to unpack their distress, regain agency, and rebuild a sense of self (El‐Abidi et al. 2024; Hartley et al. 2020; Kwame and Petrucka 2021). However, some studies have suggested that limitations in therapeutic communication and organisational support can hinder meaningful relational engagement between nurses and patients (Abraham et al. 2024; Beyene et al. 2024). Translating those ideas into psychiatric care practice may therefore be challenging, for studies have indicated that emphasising clinical procedures and risk management can limit relational engagement and, in turn, impede the realisation of recovery‐oriented care and meaningful patient participation (Chatwiriyaphong et al. 2024; Jørgensen et al. 2024; Lorien et al. 2020).

One way to integrate recovery‐oriented practice into everyday psychiatric care is through recovery‐oriented conversations (ROCs; Andvig and Biong 2014). Andvig and Biong developed ROCs as a crafted dialogic space where patients and mental health professionals (MHPs) can collaboratively unravel lived experiences, interconnect shared narratives, and chart personalised routes towards recovery. Unlike the ordinary script‐bound exchanges that populate most clinical encounters, ROCs emphasise emotional safety, a perspective of mutual understanding, and a depth of narrative beyond mere procedural compliance and symptom reduction (Maude et al. 2024; Sundet et al. 2020). ROCs may unfold spontaneously during dialogues in psychiatric settings and be deliberately integrated into formal occasions or via collaborative frameworks such as post‐incident reviews and reflective meetings (Hammervold et al. 2019), open dialogue (Buus et al. 2017), and shared decision‐making interventions (Aoki et al. 2022; Thomas et al. 2021).

Although ROCs' blend of flexibility and sustained focus on relational presence makes them especially well‐suited for mental health nursing, implementing them to promote recovery may feel difficult, which can affect their perceived authenticity and depth (Chatwiriyaphong et al. 2025; Lorien et al. 2020). That potential highlights the need to acknowledge the mentioned concerns, which may help to frame both possibilities and limitations in everyday psychiatric practice. Moreover, to date, scholarly syntheses that acknowledge ROCs as a distinct, purpose‐driven practice have been limited. Such oversight calls for an understanding about how recovery‐focused principles are enacted through conversation, as well as about the experiences and factors that influence their implementation and perceived effectiveness in psychiatric settings.

Because mental health nurses, of all mental health professionals (MHPs), spend the most one‐on‐one time with patients, they occupy a uniquely advantageous position to initiate ROCs and integrate principles of recovery into their everyday care (Macdonald 2016; McAllister et al. 2019). In support, synthesising current evidence on ROCs can guide their professional development, clinical supervision, and organisational strategies by unveiling practical approaches, flagging obstacles, and reaffirming the therapeutic role of MHPs. In doing so, ROCs may enhance recovery outcomes, foster hope and empowerment, and integrate recovery‐oriented practices throughout health services.

### 1.1 Aims

This review aims to synthesise existing research on ROCs in psychiatric settings and offer insights into their implementation, challenges, and impact on patients' recovery. In our research, those aims were guided by five research questions (RQ):

How are ROCs conceptualised in psychiatric care settings?

What approaches and strategies facilitate ROCs in psychiatric care?

How do ROCs reflect the principles of connectedness, hope, identity, meaning, and empowerment in promoting personal recovery?

How do patients and MHPs experience and perceive the outcomes of engaging in ROCs?

What factors promote or impede the implementation of ROCs in psychiatric care?

## 2 Methods

We conducted an integrative systematic review to synthesise empirical evidence on ROCs from qualitative, quantitative, and mixed‐methods studies (Whittemore and Knafl 2005). That strategy allowed integrating evidence and strengthened the overall knowledge base regarding ROCs in psychiatric settings. Our review followed the Preferred Reporting Items for Systematic Reviews and Meta‐Analyses (PRISMA) guidelines (Page et al. 2021), and its protocol was registered with the International Prospective Register of Systematic Reviews (PROSPERO). The search period, though initially set to 2019–2025 in the protocol, was subsequently broadened to also capture studies from 2015. The review's methodology was shaped by Whittemore and Knafl's (2005) five‐stage framework involving problem identification (i.e., pinpointing the review's scope and articulating the concepts), a literature search (i.e., a systematically searching for relevant studies to answer the RQs), data evaluation (i.e., assessing methodological quality and relevance), data analysis (i.e., reducing, displaying, comparing, and synthesising the data), and presentation (i.e., reporting findings with supporting evidence from primary sources).

### 2.1 Problem Identification

The integrative review was guided by the population, concept, context (PCC) framework (Peters et al. 2015), which informed the development of the RQs, inclusion criteria, and data extraction process. The *population* encompassed both patients receiving treatment and the MHPs who participated in therapeutic conversations (e.g., psychiatrists, nurses, psychologists, social workers, and peer support workers). Meanwhile, the *concept* focused on conversations that promote recovery, nurture empowerment, and prompt active participation in care, and the CHIME framework (Leamy et al. 2011) served as the lens through which components of recovery were identified and their fit with ROCs evaluated. Last, the *context* encompassed all psychiatric care settings (i.e., inpatient, outpatient, and forensic).

### 2.2 Literature Search

In collaboration with a university librarian, Promise Ezinne Ekezie performed a systematic search of the literature during 14–26 March 2025 that sought both methodological rigour and comprehensive coverage. Five databases were searched: PubMed, CINAHL, PsycINFO, Scopus, and Web of Science (see Table 1). Search terms were developed using the PCC framework, with “recovery”, “communication”, and “psychiatry” reflecting the core concept and context. Population‐related terms (e.g., “patients” and “MHPs”) were applied during the screening phase. Controlled vocabularies (e.g., MeSH, CINAHL Subject Headings, and PsycINFO Thesaurus) were used when applicable. Boolean operators, phrase searching (e.g., “mental health recovery”), and truncation (e.g., “recover*”) were employed to capture relevant variations. The search was limited to titles and abstracts (i.e., on PubMed, CINAHL, and PsycINFO) or topic fields (i.e., on Scopus and Web of Science). Limits were applied to return only English‐language, peer‐reviewed journal articles published between January 2015 and March 2025 because of increased interest in ROCs during that period. As shown in Table 1, subject filters were applied in Scopus and Web of Science to ensure relevance to healthcare, nursing, psychiatry, and psychology. Ultimately, 5298 records were identified and imported into Covidence (2025), which facilitated the removal of duplicates and supported a structured screening process.

**TABLE 1:** Overview of databases and search strategies used in the review.

Search string	Filters	Search results
“Mental Health Recovery”[Mesh] OR recover* AND “Communication”[Mesh] OR communicat* OR conversation* AND “Psychiatry”[Mesh] OR “Hospitals, Psychiatric”[Mesh] OR mental OR psychia*	*Publication years* = In the last 10 years *Language* = English *Search mode* = All fields	1743
(MH “Psychiatry” OR MH “Psychiatric Service” OR MH “Hospitals, Psychiatric”) OR (Menta* OR Psychiat*) AND MH “Recovery” OR recovery* AND (MH “Communication” OR MH “Conversation”) OR (communicat* OR convers*)	*Document type* = Full text, peer‐reviewed, academic journal article *Publication years* = 2015–2025 *Language* = English *Search mode* = Proximity *Expanders* = Apply equivalent subjects	504
DE “Recovery (Disorders)” OR recovery* AND (DE “Psychiatry” OR DE “Psychiatric Hospital Admission” OR DE “Psychiatric Units”) OR (Menta* OR Psychiat*) AND (DE “Communication” OR DE “Conversation”) OR (communicat* OR convers*)	*Document type* = Full text, peer‐reviewed, academic journal article *Publication years* = 2015–2025 *Language* = English *Search mode* = Proximity *Expanders* = Apply equivalent subjects	981
communicat* OR convers* (Topic) AND TS = (recovery) AND TS = (menta* OR psychiat*)	*Document type* = Article *Publication years* = 2015–2025; *Language*s = English ***Web of Science categories*** Psychiatry or Nursing or Public Environmental Occupational Health or Health Care Sciences Services or Psychology Clinical or Health Policy Services or Psychology Multidisciplinary or Social Work or Psychology or Substance Abuse or Psychology Applied or Paediatrics or Communication or Psychology Experimental or Psychology Developmental	773
(TITLE‐ABS‐KEY (recovery)) AND (TITLE‐ABS‐KEY (menta* OR psychiat*)) AND (TITLE‐ABS‐KEY (communicat* OR convers*))	*Document type* = Article *Publication years* = 2015–2025; *Language*s = English Limit = Journal Keyword = article *Subject areas* Medicine, Neuroscience, Nursing, Psychology, Social Sciences, Health Professions	1297

### 2.3 Selection Process

A two‐stage screening protocol was applied. First, each title and abstract were screened for eligibility by two authors; Promise Ezinne Ekezie screened all records (100%), while Alexander Rozental re‐screened 30%, Git‐Marie Ejneborn‐Looi re‐screened 30%, and Sebastian Gabrielsson re‐screened 40% of the references independently. Second, the full text of all potentially eligible articles was screened by two authors as well. Again, Promise Ezinne Ekezie screened them all, while Alexander Rozental and Sebastian Gabrielsson screened 60% and 40%, respectively. Discrepancies were resolved through discussion until consensus was reached.

**FIGURE 1:**
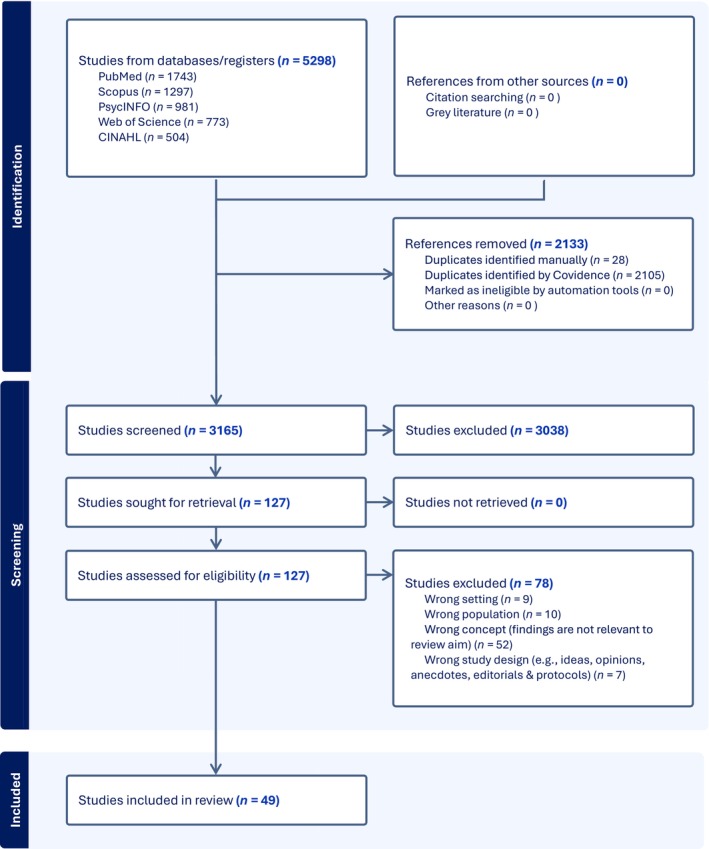
PRISMA flow diagram for article selection, adapted from Page et al. (2021).

Eligibility was defined by a set of inclusion and exclusion criteria. Included studies were peer‐reviewed empirical research (i.e., qualitative, quantitative, or mixed‐method) that focused on recovery on the basis of CHIME elements (Leamy et al. 2011) and patient–MHP conversations in psychiatric care. Opinion pieces, editorials, theoretical papers without empirical data, studies in which conversation was secondary to another intervention (e.g., medication adherence), and studies conducted outside psychiatric care were excluded. Of the initial 5298 results, 49 studies met the inclusion criteria and were included in the final synthesis. The PRISMA flow diagram in Figure 1 outlines the process.

### 2.4 Data Evaluation

The quality of the 49 studies was evaluated by using the Mixed Methods Appraisal Tool (MMAT; Hong et al. 2018) to assess all the study designs. Three authors evaluated included studies. Promise Ezinne Ekezie evaluated all 49 studies using Covidence Extraction 2.0 (Covidence 2025), which was formatted to accommodate the MMAT criteria; Alexander Rozental evaluated all quantitative and mixed‐methods studies as well as 10% of the qualitative studies; and Sebastian Gabrielsson evaluated the remaining 90%. Discrepancies were resolved through discussion until consensus was reached, and no study ended up being excluded on the grounds of quality. Table S2 lists the details of the individual quality assessments. In line with the MMAT's recommendations, no studies were graded on quality; instead, each study received a narrative comment reflecting its methodological rigour, as shown in the overall appraisal summary in Table S1.

### 2.5 Data Extraction

Data were extracted using Covidence Extraction 2.0 (Covidence 2025), primarily because it allows developing custom templates that capture relevant components such as study design, setting, aims, key findings, and elements aligned with the PCC framework. Data were reduced by extracting and organising findings according to the five RQs. The included studies were grouped by methodology, and key elements (e.g., conceptualisations, approaches, CHIME components, experiential findings, and barriers/facilitators) were summarised into a table of definitions and attributes. The table subsequently informed a data‐display mapping strategy that allowed charting outcome relationships and assigning each barrier and facilitator across settings. Promise Ezinne Ekezie performed the extraction process independently, after which each co‐author re‐examined the extracted data to ensure its accuracy and relevance to the review's aim, as detailed in Table S1.

### 2.6 Data Analysis

Given the methodological diversity of the included studies and the scope of the RQs, we adopted a narrative synthesis approach that followed Popay et al.'s (2005) guidelines. Promise Ezinne Ekezie independently conducted the analysis, after which all authors participated in reviewing and refining the findings to achieve rigour and consensus. Both inductive and deductive approaches were employed; the inductive approach was applied to RQ1, RQ2, RQ4, and RQ5, which began by organising raw data to identify patterns and ideas, whereas deductive approaches began by building on existing theory to test specific data (Barroga et al. 2023), guided by the CHIME framework (Leamy et al. 2011). The deductive approach was applied to RQ3 by linking each finding to the five CHIME elements and their respective indicators: connectedness (i.e., support from others, peer support and support groups, community involvement, and relationships), hope (i.e., belief in recovery, motivation to change, positive thinking and optimism, and valuing success and achievement), identity (i.e., rebuilding and/or redefining a positive sense of self, self‐acceptance, overcoming stigma, and personal growth) meaning (i.e., meaningful life roles, spirituality and existential reflection, quality of life, social goals and responsibilities), and empowerment (i.e., personal responsibility, control over life, focusing on strengths, and Self‐management of mental health).

To ensure rigour, we compared and triangulated data across sources; identified recurring patterns, contradictions, and explanatory factors; and wove together qualitative and quantitative strands to determine convergence, complementarity, or divergence. Subgroup analyses examined how experiences, settings, conversational structures, and outcomes might have shaped the findings. The conclusions were derived by synthesising higher‐order themes, a process underpinned by investigator triangulation, a thorough audit trail, and sensitivity analyses. The conclusions were subsequently reviewed by a service user reference group (SURG)—that is, three individuals with lived experience of psychiatric care who provided their insights on the synthesis from the perspective of patients.

#### 2.6.1 Researcher Positionality

Promise Ezinne Ekezie is a mental health nurse and doctoral candidate, whereas all other co‐authors contributed a mix of clinical and scholarly expertise spanning mental health nursing, psychology, and psychiatry. Our combined background in therapeutic engagement, recovery‐oriented care, and diverse psychiatric settings informed the synthesis. Reflexivity was maintained throughout, and input from SURG ensured that the perspective of lived experience remained central to the review (Völlm et al. 2017).

## 3 Results

This review synthesises existing research on ROCs by exploring their implementation, experiences, and influencing factors in psychiatric care. In this section, we provide a descriptive summary of the studies, an overview of the evidence base (see Table S1), and subsequent syntheses guided by the RQs presented thematically into five themes: conceptualising ROCs (see Table 2), facilitating ROCs in practice (see Table 3), the alignment of ROCs with the CHIME framework (see Table 4), experiences and perceived outcomes of ROCs (see Table 5), and implementing ROCs in psychiatric care (see Table 6).

**TABLE 2:** Overview of the conceptualisation of recovery‐oriented conversations (ROCs) in psychiatric care.

Theme	Descriptor (*n*)	Supporting studies	Illustrative quotations and evidence
ROCs as relational practices sustained through everyday contact	Relational anchoring (28 studies)	Banfield and Forbes (2018), Bradley et al. (2021), Donaghay‐Spire et al. (2016), Faith et al. (2023), Forchuk et al. (2021), Hristodoulidis et al. (2022), Karbouniaris et al. (2022), Keefe et al. (2020), Kehoe et al. (2023), Kirkegaard Thomsen et al. (2024), Lauzier‐Jobin and Houle (2024), Molin et al. (2018), Molin et al. (2020), Pelto‐Piri et al. (2019), Pfeiffer et al. (2019), Priebe et al. (2018), Prytz et al. (2019), Rooney et al. (2016), Scheirich et al. (2024), Sellin et al. (2018), Selvin et al. (2021), Solomon et al. (2021), Twamley et al. (2021), Vandewalle et al. (2019), Walde et al. (2023), Waldemar et al. (2019), Wallace et al. (2016), Whittle et al. (2024)	“Caring describes the healing words and human contact … through interactions with staff and between patients, was desired and highly valued by inpatient participants” (Bradley et al. 2021, 922) “Getting to truly know people … developing these really rich relationships” (Faith et al. 2023, 2186)
	Supportive presence (48 studies)	Banfield and Forbes (2018), Bradley et al. (2021), Carmel et al. (2018), Donaghay‐Spire et al. (2016), Eiroa‐Orosa and Incera‐Rosas (2025), Faith et al. (2023), Forchuk et al. (2021), Hammervold et al. (2022), Horgan et al. (2021), Howell et al. (2023), Hristodoulidis et al. (2022), Hyde et al. (2015), Igarashi et al. (2024), Isobel (2019), Jørgensen et al. (2022), Karbouniaris et al. (2022), Keefe et al. (2020), Kehoe et al. (2023), Kidd et al. (2015), Kirkegaard Thomsen et al. (2024), Klevan et al. (2024), Lauzier‐Jobin and Houle (2024), Molin et al. (2018), Molin et al. (2020), Okumura and Katsuki (2024), Pelto‐Piri et al. (2019), Pfeiffer et al. (2019), Priebe et al. (2018), Prytz et al. (2019), Raitakari et al. (2018), Reed et al. (2018), Reinius et al. (2023), Rooney et al. (2016), Scheirich et al. (2024), Sellin et al. (2018), Sellin et al. (2019), Selvin et al. (2021), Shue et al. (2023), Solomon et al. (2021), Twamley et al. (2021), Vandewalle et al. (2019), Walde et al. (2023), Waldemar et al. (2019), Wallace et al. (2016), Whittle et al. (2024), Wong et al. (2019), Zetterström et al. (2023), van Lankeren et al. (2020)	“The psychologist appeared and said here's a space to talk, we're all listening. And I think it just makes such a difference” (Donaghay‐Spire et al. 2016, 473) “Being able to sit with pain … not try to make it better” (Faith et al. 2023, 2184)
	Emotion‐focused interaction (19 studies)	Bradley et al. (2021), Coelho et al. (2024), Donaghay‐Spire et al. (2016), Faith et al. (2023), Hammervold et al. (2022), Horgan et al. (2021), Isobel (2019), Keefe et al. (2020), Kehoe et al. (2023), Kirkegaard Thomsen et al. (2024), Priebe et al. (2018), Reed et al. (2018), Scheirich et al. (2024), Sellin et al. (2018), Sellin et al. (2019), Solomon et al. (2021), Twamley et al. (2021), Vandewalle et al. (2019), van Lankeren et al. (2020)	“Be calm”; “Nurses should use a softer tone of voice” (Coelho et al. 2024, 6) “I couldn't talk about it at the start but I slowly … crying” (Donaghay‐Spire et al. 2016, 473)
	Rhythmic engagement (12 studies)	Banfield and Forbes (2018), Forchuk et al. (2021), Horgan et al. (2021), Kehoe et al. (2023), Klevan et al. (2024), Molin et al. (2018), Raitakari et al. (2018), Reed et al. (2018), Sellin et al. (2018), Sellin et al. (2019), Shue et al. (2023), Zetterström et al. (2023)	“It's like ‘OK, you're struggling with that, what can we do to, you know, change your life, tweak it in ways that you can do that better’” (Banfield and Forbes 2018, 7)
ROCs as shared spaces for structuring talk and direction	Clarity‐oriented communication (15 studies)	Bradley et al. (2021), Hyde et al. (2015), Kidd et al. (2015), Kirkegaard Thomsen et al. (2024), Lauzier‐Jobin and Houle (2024), Molin et al. (2020), Pfeiffer et al. (2019), Reinius et al. (2023), Scheirich et al. (2024), Sellin et al. (2018), Twamley et al. (2021), Vandewalle et al. (2019), Wong et al. (2019), Zetterström et al. (2023), van Lankeren et al. (2020)	“The division of anxiety into three levels … enabled nurses and patients to start from a common structure when talking about the patient's anxiety” (Zetterström et al. 2023, 904)
	Voice‐centred exchange (18 studies)	Banfield and Forbes (2018), Bradley et al. (2021), Donaghay‐Spire et al. (2016), Hammervold et al. (2022), Horgan et al. (2021), Hyde et al. (2015), Isobel (2019), Jørgensen et al. (2022), Keefe et al. (2020), Kirkegaard Thomsen et al. (2024), Lauzier‐Jobin and Houle (2024), Pfeiffer et al. (2019), Scheirich et al. (2024), Sellin et al. (2019), Twamley et al. (2021), Waldemar et al. (2019), Wallace et al. (2016), Zetterström et al. (2023)	“Open dialogue is where professionals use active listening” (Jørgensen et al. 2022, 168) “The role in this whole relationship was really to listen to her” (Lauzier‐Jobin and Houle 2024, 73). “I was able to explain myself without interruption… they realised they had to listen to me…” (Hammervold et al. 2022, 477)
	Choice‐driven communication (10 studies)	Faith et al. (2023), Igarashi et al. (2024), Isobel (2019), Okumura and Katsuki (2024), Raitakari et al. (2018), Reinius et al. (2023), Scheirich et al. (2024), Sellin et al. (2018), Shue et al. (2023), Waldemar et al. (2019)	“It can be interpreted from the interaction that for the “recovery in” process, a client who is too adapting and pliable is a problem, because one aim of the RIM is to restore a client's self‐determination and right to make decisions according to his/her own will” (Raitakari et al. 2018, 11)
ROCs as convers‐ations grounded in personal and social realities	Context‐responsive talk (8 studies)	Bradley et al. (2021), Isobel (2019), Jørgensen et al. (2022), Klevan et al. (2024), Selvin et al. (2021), Shue et al. (2023), Wong et al. (2019), Zetterström et al. (2023)	“Conversation … strives to understand the circumstances… what they would like to get out of the hospitalisation.” (Shue et al. 2023, 288)
	Story‐linked dialogue (8 studies)	Carmel et al. (2018), Hyde et al. (2015), Karbouniaris et al. (2022), Kidd et al. (2015), Klevan et al. (2024), Rooney et al. (2016), Sellin et al. (2018), Wallace et al. (2016)	“Let them tell their story … being listened to … probably the most important part” (Hyde et al. 2015, 70)

**TABLE 3:** Overview of the approaches and strategies for facilitating recovery‐oriented conversations.

Theme	Descriptor (*n*)	Supporting studies	Illustrative quotations and evidence
Deepening dialogue through emotional and narrative work	Reflective and meaning‐making dialogue (44 studies)	Banfield and Forbes (2018), Bradley et al. (2021), Carmel et al. (2018), Coelho et al. (2024), Donaghay‐Spire et al. (2016), Eiroa‐Orosa and Incera‐Rosas (2025), Faith et al. (2023), Forchuk et al. (2021), Hammervold et al. (2022), Horgan et al. (2021), Howell et al. (2023), Hristodoulidis et al. (2022), Hyde et al. (2015), Igarashi et al. 2024, Isobel (2019), Jørgensen et al. (2022), Karbouniaris et al. (2022), Keefe et al. (2020), Kehoe et al. (2023), Kidd et al. (2015), Kirkegaard Thomsen et al. (2024), Klevan et al. (2024), Lauzier‐Jobin and Houle (2024), Molin et al. (2018), Molin et al. (2020), Pelto‐Piri et al. (2019), Pfeiffer et al. (2019), Priebe et al. (2018), Raitakari et al. (2018), Reed et al. (2018), Sellin et al. (2018), Sellin et al. (2019), Selvin et al. (2021), Shue et al. (2023), Solomon et al. (2021), Twamley et al. (2021), Vandewalle et al. (2019), van Lankeren et al. (2020), Walde et al. (2023), Waldemar et al. (2019), Wallace et al. (2016), Whittle et al. (2024), Wong et al. (2019), Zetterström et al. (2023)	“Talking with consumers has helped me see that [resilience] exists” (Kidd et al. 2015, 43) “Let's talk about it together and let's think together” (Faith et al. 2023, 2184)
	Emotional expression and validation (13 studies)	Banfield and Forbes (2018), Coelho et al. (2024), Donaghay‐Spire et al. (2016), Forchuk et al. (2021), Horgan et al. (2021), Howell et al. (2023), Hyde et al. (2015), Karbouniaris et al. (2022), Kehoe et al. (2023), Lauzier‐Jobin and Houle (2024), Okumura and Katsuki (2024), Pelto‐Piri et al. (2019), Sellin et al. (2018)	“When we're sad, sometimes it's best to express our emotions to someone willing to listen” (Coelho et al. 2024, 6)
Dialogically co‐constructing recovery‐oriented pathways	Collaborative planning and decision‐making (20 studies)	Banfield and Forbes (2018), Hammervold et al. (2022), Igarashi et al. (2024), Jørgensen et al. (2022), Karbouniaris et al. (2022), Keefe et al. (2020), Kehoe et al. (2023), Kidd et al. (2015), Molin et al. (2018), Okumura and Katsuki (2024), Pfeiffer et al. (2019), Priebe et al. (2018), Reed et al. (2018), Selvin et al. (2021), Shue et al. (2023), Walde et al. (2023), Waldemar et al. (2019), Wallace et al. (2016), Whittle et al. (2024), Wong et al. (2019)	“Collaborative goal‐setting and recovery planning received 100% positive responses at mid‐ and endpoint respectively” (Banfield and Forbes 2018, 5) “Worked out a mutual agreement on how to handle situations” (Hammervold et al. 2022, 478) “We just talk about getting me out of here and how I'm gonna do…and we talk about how I need to stop the habits I had, so that has been helpful” (Keefe et al. 2020, 204)
	Inclusive and equal participation (5 studies)	Eiroa‐Orosa and Incera‐Rosas (2025), Jørgensen et al. (2022), Kidd et al. (2015), Selvin et al. (2021), Wallace et al. (2016)	“Everyone's opinions are equally heard” (Jørgensen et al. 2022, 168) “Patient participation demanded mutual engagement through active communication and that staff should encourage the patient to be active and involved” (Selvin et al. 2021, 463)
	Use of tools and structured aids (6 studies)	Igarashi et al. (2024), Kehoe et al. (2023), Kidd et al. (2015), Pfeiffer et al. (2019), Reed et al. (2018), Wallace et al. (2016)	“Conversations started by referring to SHARE sheets” (Igarashi et al. (2024), 4)
	Peer and lived experience integration (3 studies)	Kehoe et al. (2023), Kidd et al. (2015), Pfeiffer et al. (2019)	“The peer has been in my situation, so it was really easy to relate.” (Pfeiffer et al. 2019, 366).
	Agency and self‐direction support (3 studies)	Okumura and Katsuki (2024), Priebe et al. (2018), Zetterström et al. (2023)	“It could be a strengthening ‘aha’ experience for the patient to discover that they could choose to cope with their anxiety in different ways” (Zetterström et al. 2023, 905)
Staying present to support conversation	Supportive communication techniques (18 studies)	Banfield and Forbes (2018), Coelho et al. (2024), Donaghay‐Spire et al. (2016), Eiroa‐Orosa and Incera‐Rosas (2025), Hammervold et al. (2022), Horgan et al. (2021), Hyde et al. (2015), Jørgensen et al. (2022), Keefe et al. (2020), Kirkegaard Thomsen et al. (2024), Lauzier‐Jobin and Houle (2024), Pfeiffer et al. (2019), Priebe et al. (2018), Sellin et al. (2018), Solomon et al. (2021), Twamley et al. (2021), Vandewalle et al. (2019), van Lankeren et al. (2020)	“It is crucial to have that active listening, empathic listening … listen with their heart, eyes and all their senses” (Horgan et al. 2021, 141) “Listening and the contract that I managed to make with her gave her the opportunity to walk the path of healing” (Kirkegaard Thomsen et al. 2024, 22)
	Therapeutic relationship building (10 studies)	Banfield and Forbes (2018), Donaghay‐Spire et al. (2016), Isobel (2019), Jørgensen et al. (2022), Keefe et al. (2020), Molin et al. (2020), Priebe et al. (2018), Sellin et al. (2018), Shue et al. (2023), Walde et al. (2023)	“Relationships built during TT had a basis of humanity and sharing” (Molin et al. 2020, 1197)
	Informal and everyday talk (3 studies)	Bradley et al. (2021), Keefe et al. (2020), Klevan et al. (2024)	“Talking about healthy bread, fruits … all those little, silly things” (Klevan et al. 2024, 1126).
	Relational grounding (5 studies)	Jørgensen et al. (2022), Lauzier‐Jobin and Houle (2024), Pfeiffer et al. (2019), Wallace et al. (2016), van Lankeren et al. (2020)	“We're no longer in professional mode, we're in partner mode” (Lauzier‐Jobin and Houle 2024, 75).
Designing conditions that invite conversation	Environment‐supported communication (13 studies)	Coelho et al. (2024), Donaghay‐Spire et al. (2016), Eiroa‐Orosa and Incera‐Rosas (2025), Hyde et al. (2015), Jørgensen et al. (2022), Karbouniaris et al. (2022), Keefe et al. (2020), Klevan et al. (2024), Lauzier‐Jobin and Houle (2024), Molin et al. (2020), Priebe et al. (2018), Sellin et al. (2019), Solomon et al. (2021)	“TT brought about a change in the atmosphere on the units, with fellowship and safety developing” (Molin et al. 2020, 1197) “Giving the patient space to suffer in communion with a compassionate fellow human being” (Priebe et al. 2018, 860) “It is a really positive environment… programs allow you to explore different hobbies” (Kehoe et al. 2023, 7)
	Cultural and contextual sensitivity (3 studies)	Bradley et al. (2021), Shue et al. (2023), Solomon et al. (2021)	“Use of shared language and song can create strong threads of connection, cultural meaning, and sense of safety” (Solomon et al. 2021, 968)

Recovery‐oriented group dialogue (4 studies)	Jørgensen et al. (2022), Kehoe et al. (2023), Shue et al. (2023), Walde et al. (2023)	“Recovery group … covered several recovery relevant topics, but also used these meetings as space to discuss current issues” (Walde et al. 2023, 7) “Group programming daily tailored to meet veteran needs” (Shue et al. 2023, 291)

**TABLE 4:** Overview of the reflection of CHIME principles in recovery‐oriented conversations.

CHIME framework	Indicator (*n*)	Supporting studies	Illustrative quotations and evidence
Connectedness	Support from others (29 studies)	Banfield and Forbes (2018), Bradley et al. (2021), Carmel et al. (2018), Coelho et al. (2024), Donaghay‐Spire et al. (2016), Eiroa‐Orosa and Incera‐Rosas (2025), Forchuk et al. (2021), Hammervold et al. (2022), Howell et al. (2023), Hristodoulidis et al. (2022), Igarashi et al. (2024), Jørgensen et al. (2022), Karbouniaris et al. (2022), Keefe et al. (2020), Klevan et al. (2024), Pelto‐Piri et al. (2019), Pfeiffer et al. (2019), Priebe et al. (2018), Reed et al. (2018), Reinius et al. (2023), Rooney et al. (2016), Sellin et al. (2019), Selvin et al. (2021), Shue et al. (2023), Vandewalle et al. (2019), Wallace et al. (2016), Whittle et al. (2024), Wong et al. (2019), van Lankeren et al. (2020)	“Caring, expressed through interactions with staff and between patients, was desired and highly valued by inpatient participants” (Bradley et al. 2021, 922) “Professionals function as mediators in negotiations between users and others/social arenas; ‘translating’ between the parties in order to promote mutual understanding” (Reed et al. 2018, 819) “Sit down with me so I feel you're here for me” (Pelto‐Piri et al. 2019, 5)
	Relationships (22 studies)	Bradley et al. (2021), Carmel et al. (2018), Donaghay‐Spire et al. (2016), Faith et al. (2023), Forchuk et al. (2021), Hammervold et al. (2022), Howell et al. (2023), Igarashi et al. (2024), Karbouniaris et al. (2022), Keefe et al. (2020), Lauzier‐Jobin and Houle (2024), Pfeiffer et al. (2019), Priebe et al. (2018), Reinius et al. (2023), Rooney et al. (2016), Sellin et al. (2019), Twamley et al. (2021), Vandewalle et al. (2019), Wallace et al. (2016), Whittle et al. (2024), Wong et al. (2019), van Lankeren et al. (2020)	“We get to know each other beyond the diagnosis and why you are here. … It is more like a meeting between two people” (Reinius et al. 2023, 763) “Like I've got family here to talk to, to ask them” (Bradley et al. 2021, 922) “We hit it off, like I think just in terms of personality, interests, um background experience, everything, it's a perfect match” (Forchuk et al. 2021, 557)
	Community involvement (8 studies)	Carmel et al. (2018), Jørgensen et al. (2022), Keefe et al. (2020), Klevan et al. (2024), Lauzier‐Jobin and Houle (2024), Reed et al. (2018), Reinius et al. (2023), Shue et al. (2023)	“We have a community meeting every single morning. And so, we view, of course, the veteran as part of the interdisciplinary team”: (Shue et al. 2023, 287)
	Peer support and support groups (8 studies)	Donaghay‐Spire et al. (2016), Forchuk et al. (2021), Hyde et al. (2015), Lauzier‐Jobin and Houle (2024), Pfeiffer et al. (2019), Rooney et al. (2016), Twamley et al. (2021), Whittle et al. (2024)	“It was like talking to a friend instead of a counsellor” (Pfeiffer et al. 2019, 366)
Hope	Belief in recovery (25 studies)	Banfield and Forbes (2018), Bradley et al. (2021), Carmel et al. (2018), Coelho et al. (2024), Donaghay‐Spire et al. (2016), Forchuk et al. (2021), Hammervold et al. (2022), Horgan et al. (2021), Howell et al. (2023), Hristodoulidis et al. (2022), Isobel (2019), Jørgensen et al. (2022), Karbouniaris et al. (2022), Kehoe et al. (2023), Kidd et al. (2015), Lauzier‐Jobin and Houle (2024), Molin et al. (2020), Pelto‐Piri et al. (2019), Priebe et al. (2018), Reed et al. (2018), Rooney et al. (2016), Scheirich et al. (2024), Sellin et al. (2018, 2019), Vandewalle et al. (2019)	“We've come a really long way because in January I didn't feel there was no hope at all. But I can talk about it” (Donaghay‐Spire et al. 2016, 474) “The professionals must listen to the hopes that users have and to show confidence in a user when it comes to fulfilling his or her hopes” (Jørgensen et al. 2022, 167) “I'm going to lend you my hope until you build up your own and, after that, I'll take it back” (Lauzier‐Jobin and Houle 2024, 74)
	Motivation to change (14 studies)	Bradley et al. (2021), Faith et al. (2023), Forchuk et al. (2021), Igarashi et al. (2024), Keefe et al. (2020), Kehoe et al. (2023), Lauzier‐Jobin and Houle (2024), Okumura and Katsuki (2024), Prytz et al. (2019), Reed et al. (2018), Scheirich et al. (2024), Sellin et al. (2018), Vandewalle et al. (2019), van Lankeren et al. (2020)	“Patients who wanted jobs consulted doctors about the job‐hunting process” (Igarashi et al. 2024, 4)
	Positive thinking and optimism (20 studies)	Bradley et al. (2021), Eiroa‐Orosa and Incera‐Rosas (2025), Hammervold et al. (2022), Hyde et al. (2015), Isobel (2019), Jørgensen et al. (2022), Karbouniaris et al. (2022), Keefe et al. (2020), Kidd et al. (2015), Klevan et al. (2024), Molin et al. (2018, 2020), Pelto‐Piri et al. (2019), Priebe et al. (2018), Raitakari et al. (2018), Reinius et al. (2023), Scheirich et al. (2024), Sellin et al. (2019), Vandewalle et al. (2019), van Lankeren et al. (2020)	“Hope and optimism were discussed as having something positive to look forward to in one's life and for maintaining optimism in the recovery process” (Jørgensen et al. 2022, 167) “I always try to listen for sparkles of hope in a conversation such as things they like or used to like, hobbies, things they are very passionate about.” (Vandewalle et al. 2019, 2873)
	Valuing success and achievement (6 studies)	Faith et al. (2023), Jørgensen et al. (2022), Kidd et al. (2015), Lauzier‐Jobin and Houle (2024), Molin et al. (2020), Reinius et al. (2023)	“Participants also believed that Daily Talks accelerated their recovery” (Reinius et al. 2023, 765) “They continue to find ways to make a life that matters to them and to see them doing things again and getting back to life—that really is fulfilling” (Faith et al. 2023, 2186)
Identity	Rebuilding and/or redefining a positive sense of self (28 studies)	Banfield and Forbes (2018), Bradley et al. (2021), Donaghay‐Spire et al. (2016), Eiroa‐Orosa and Incera‐Rosas (2025), Faith et al. (2023), Forchuk et al. (2021), Hammervold et al. (2022), Horgan et al. (2021), Hyde et al. (2015), Igarashi et al. (2024), Jørgensen et al. (2022), Kehoe et al. (2023), Kidd et al. (2015), Kirkegaard Thomsen et al. (2024), Klevan et al. (2024), Lauzier‐Jobin and Houle (2024), Molin et al. (2018), Molin et al. (2020), Okumura and Katsuki (2024), Pfeiffer et al. (2019), Raitakari et al. (2018), Reed et al. (2018), Reinius et al. (2023), Rooney et al. (2016), Scheirich et al. (2024), Sellin et al. (2018), Sellin et al. (2019), Selvin et al. (2021)	“I'm a human being, I'm an individual, I am a person, and each person is going to present differently” (Horgan et al. 2021, 142) “The staff explained that they left their specific roles—an ill patient and a nurse—and gave more of themselves as persons” (Molin et al. 2020, 1196)
	Self‐acceptance (20 studies)	Bradley et al. (2021), Coelho et al. (2024), Donaghay‐Spire et al. (2016), Faith et al. (2023), Forchuk et al. (2021), Hammervold et al. (2022), Hyde et al. (2015), Igarashi et al. (2024), Karbouniaris et al. (2022), Keefe et al. (2020), Klevan et al. (2024), Lauzier‐Jobin and Houle (2024), Pfeiffer et al. (2019), Priebe et al. (2018), Raitakari et al. (2018), Sellin et al. (2018), Sellin et al. (2019), Selvin et al. (2021), Solomon et al. (2021), Twamley et al. (2021)	“She was ‘allowed to have a voice and became seen and believed,’ consequently, she perceived her experiences and views as having been acknowledged” (Hammervold et al. 2022, 477)
	Overcoming stigma (14 studies)	Carmel et al. (2018), Eiroa‐Orosa and Incera‐Rosas 2025, Hammervold et al. (2022), Horgan et al. (2021), Isobel (2019), Jørgensen et al. (2022), Karbouniaris et al. (2022), Kidd et al. (2015), Lauzier‐Jobin and Houle (2024), Priebe et al. (2018), Reed et al. (2018), Rooney et al. (2016), Solomon et al. (2021), Twamley et al. (2021)	“Once she came to me and told me that I am not my depression, even though I suffer from depression. She just flipped my perspective, it was such an eye‐opener to me!” (Karbouniaris et al. 2022, 30) “She isn't patronising and she doesn't talk illness. … She's never asked me what my diagnoses is because it doesn't really matter to her” (Rooney et al. 2016, 162)
	Personal growth (14 studies)	Jørgensen et al. (2022), Keefe et al. (2020), Kehoe et al. (2023), Kirkegaard Thomsen et al. (2024), Lauzier‐Jobin and Houle (2024), Molin et al. (2018), Molin et al. (2020), Okumura and Katsuki (2024), Pelto‐Piri et al. (2019), Prytz et al. (2019), Reinius et al. (2023), Scheirich et al. (2024), Sellin et al. (2018), Shue et al. (2023)	“One participant described how controlling the content of Daily Talks could lead to patients feeling like they are in control of their own healthcare and health” (Reinius et al. 2023, 764) “If you want them to be competent, emphasise what they're good at and they'll work even harder” (Lauzier‐Jobin and Houle 2024, 74)
Meaning	Meaningful life roles (18 studies)	Banfield and Forbes (2018), Carmel et al. (2018), Donaghay‐Spire et al. (2016), Faith et al. (2023), Jørgensen et al. (2022), Karbouniaris et al. (2022), Kehoe et al. (2023), Kidd et al. (2015), Kirkegaard Thomsen et al. (2024), Lauzier‐Jobin and Houle (2024), Okumura and Katsuki (2024), Pfeiffer et al. (2019), Raitakari et al. (2018), Reed et al. (2018), Rooney et al. (2016), Shue et al. (2023), Waldemar et al. (2019), Wallace et al. (2016)	“Stimulated them to construct their own narrative, find meanings and arrive at deeper insights” (Karbouniaris et al. 2022, 30) “Meaning and purpose were discussed as living according to the values that mean something to that person” (Jørgensen et al. 2022, 168) “Partners in Recovery is like an advocate. They are, they can speak for you, you tell them what you want and they know exactly where to go, what to do to get it done” (Banfield and Forbes 2018, 7)
	Social goals and responsibilities (30 studies)	Banfield and Forbes (2018), Bradley et al. (2021), Carmel et al. (2018), Coelho et al. (2024), Eiroa‐Orosa and Incera‐Rosas (2025), Forchuk et al. (2021), Horgan et al. (2021), Howell et al. (2023), Hyde et al. (2015), Isobel (2019), Jørgensen et al. (2022), Kehoe et al. (2023), Kidd et al. (2015), Lauzier‐Jobin and Houle (2024), Molin et al. (2018), Molin et al. (2020), Pelto‐Piri et al. (2019), Prytz et al. (2019), Reed et al. (2018), Rooney et al. (2016), Sellin et al. (2019), Selvin et al. (2021), Shue et al. (2023), Twamley et al. (2021), Walde et al. (2023), Waldemar et al. (2019), Wallace et al. (2016), Whittle et al. (2024), Zetterström et al. (2023), van Lankeren et al. (2020)	“Your say in what was important”—‘95% (midpoint)’, ‘93% (endpoint)’ (Banfield and Forbes 2018, 6) “Inpatients and ARG agreed that the Activity Centre is a focus for care and healing, encouraging culturally appropriate activity and interaction” (Bradley et al. 2021, 922) “He does respond well to structured activity, for example, the walking group, getting out and about” (Whittle et al. 2024, 8)
	Quality of life (27 studies)	Bradley et al. (2021), Donaghay‐Spire et al. (2016), Faith et al. (2023), Hammervold et al. (2022), Hristodoulidis et al. (2022) Igarashi et al. (2024), Isobel (2019), Jørgensen et al. (2022) Keefe et al. (2020), Kehoe et al. (2023), Klevan et al. (2024), Kirkegaard Thomsen et al. (2024), Molin et al. (2020), Priebe et al. (2018), Prytz et al. (2019), Raitakari et al. (2018), Reinius et al. (2023), Scheirich et al. (2024), Sellin et al. (2018), Sellin et al. (2019), Twamley et al. (2021), Vandewalle et al. (2019), Walde et al. (2023), Whittle et al. (2024), Wong et al. (2019), Zetterström et al. (2023), van Lankeren et al. (2020)	“Especially the nurses with more than 10 years of working experience stressed the importance of taking assessment as part of an open conversation, informing and discussing the application of procedures with patients (e.g., time of observations) and explaining to patients how procedures contribute to good and safe care” (Vandewalle et al. 2019, 2872) “Make sure the nurses and social workers and AMHWs are letting us know that they're there when we need to talk to them. And y'know just letting us know when we come in—and even keep on telling us—‘We're here’” (Bradley et al. 2021, 924) “Programs really allow you to explore different hobbies and opens you up to new things” (Kehoe et al. 2023, 7)
	Spirituality (6 studies)	Hyde et al. (2015), Jørgensen et al. (2022), Keefe et al. (2020), Pfeiffer et al. (2019), Priebe et al. (2018), Solomon et al. (2021)	“Creating space for sharing stories, meaning making, as well as the processing of grief and loss” (Solomon et al. 2021, 969)
Empowerment	Control over life (44 studies)	Banfield and Forbes (2018), Bradley et al. (2021), Carmel et al. (2018), Donaghay‐Spire et al. (2016), Eiroa‐Orosa and Incera‐Rosas (2025), Faith et al. (2023), Forchuk et al. (2021), Hammervold et al. (2022), Horgan et al. (2021), Hristodoulidis et al. (2022), Hyde et al. (2015), Igarashi et al. (2024), Isobel (2019), Jørgensen et al. (2022), Karbouniaris et al. (2022), Keefe et al. (2020), Kehoe et al. (2023), Kidd et al. (2015), Klevan et al. (2024), Kirkegaard Thomsen et al. (2024), Lauzier‐Jobin and Houle (2024), Molin et al. (2018), Okumura and Katsuki (2024), Priebe et al. (2018), Prytz et al. (2019), Raitakari et al. (2018), Reed et al. (2018), Reinius et al. (2023), Rooney et al. (2016), Scheirich et al. (2024), Sellin et al. (2018), Sellin et al. (2019), Selvin et al. (2021), Shue et al. (2023), Solomon et al. (2021), Twamley et al. (2021), Vandewalle et al. (2019), Walde et al. (2023), Waldemar et al. (2019), Wallace et al. (2016), Whittle et al. (2024), Wong et al. (2019), Zetterström et al. (2023), van Lankeren et al. (2020)	“Sense of control”—‘90% (midpoint)’, ‘93% (endpoint)’ (Banfield and Forbes 2018, 6) “I've put trust in them [nurse and psychologist]. … I'd say support was a big thing. Talking about it, and just helping along the way” (Donaghay‐Spire et al. 2016, 473) “Patients could also initiate negotiations by proposing to accept the medical treatment on conditions of transferral or discharge” (Waldemar et al. 2019, 324)
	Self‐management of mental health (14 studies)	Banfield and Forbes (2018), Faith et al. (2023), Horgan et al. (2021), Jørgensen et al. (2022), Kehoe et al. (2023), Kirkegaard Thomsen et al. (2024), Lauzier‐Jobin and Houle (2024), Molin et al. (2020), Okumura and Katsuki (2024), Pfeiffer et al. (2019), Sellin et al. (2019), Twamley et al. (2021), Zetterström et al. (2023), van Lankeren et al. (2020)	“This is what I think about the anxiety communication notes: they improve the patient's self‐reflection about their anxiety and how they can lower their anxiety level” (Zetterström et al. 2023, 905)
	Focusing on strengths (15 studies)	Banfield and Forbes (2018), Coelho et al. (2024), Donaghay‐Spire et al. (2016), Faith et al. (2023), Forchuk et al. (2021), Howell et al. (2023), Hristodoulidis et al. (2022), Karbouniaris et al. (2022), Kirkegaard Thomsen et al. (2024), Lauzier‐Jobin and Houle (2024), Molin et al. (2020), Raitakari et al. (2018), Solomon et al. (2021), Wallace et al. (2016), Whittle et al. (2024)	“The good ones try and encourage you in other areas … I think it makes you feel more positive” (Whittle et al. 2024, 6)
	Personal responsibility (10 studies)	Carmel et al. (2018), Eiroa‐Orosa and Incera‐Rosas (2025), Faith et al. (2023), Isobel (2019), Keefe et al. (2020), Okumura and Katsuki (2024), Pelto‐Piri et al. (2019), Scheirich et al. (2024), Sellin et al. (2018), Twamley et al. (2021)	“Participants stressed the importance that you, as a patient, are taking responsibility for your own rehabilitation” (Pelto‐Piri et al. 2019, 6) “You can't really speak up … or else they might get mad. … I don't wanna offend nobody” (Keefe et al. 2020, 202)

Abbreviation: CHIME = connectedness, hope, identity, motivation, and empowerment.

**TABLE 5:** Overview of experiences and perceived outcomes of recovery‐oriented conversations.

Theme	Descriptor (*n*)	Supporting studies	Illustrative quotations and evidence
Feeling human in the eyes of others	Being treated as a person (28 studies)	Bradley et al. (2021), Coelho et al. (2024), Hammervold et al. (2022), Horgan et al. (2021), Hyde et al. (2015), Isobel (2019), Jørgensen et al. (2022), Karbouniaris et al. (2022), Kehoe et al. (2023), Kirkegaard Thomsen et al. (2024), Lauzier‐Jobin and Houle (2024), Molin et al. (2020), Molin et al. (2018), Pelto‐Piri et al. (2019), Pfeiffer et al. (2019), Priebe et al. (2018), Prytz et al. (2019), Raitakari et al. (2018), Reinius et al. (2023), Rooney et al. (2016), Scheirich et al. (2024), Sellin et al. (2018), Sellin et al. (2019), Selvin et al. (2021), Shue et al. (2023), Twamley et al. (2021), Wallace et al. (2016), Wong et al. (2019)	“The ones that treat me like a human being make a big difference” (Isobel 2019, 111) “I came here as a person. … They've classified me as schizophrenic” (Hyde et al. 2015, 70) “To be respected and regarded as a human being … strengthens one's dignity” (Priebe et al. 2018, 861)
	Connection that feels mutual (19 studies)	Eiroa‐Orosa and Incera‐Rosas (2025), Horgan et al. (2021), Klevan et al. (2024), Lauzier‐Jobin and Houle (2024), Molin et al. (2020), Molin et al. (2018), Pelto‐Piri et al. (2019), Pfeiffer et al. (2019), Priebe et al. (2018), Prytz et al. (2019), Raitakari et al. (2018), Reinius et al. (2023), Scheirich et al. (2024), Sellin et al. (2018), Selvin et al. (2021), Twamley et al. (2021), Waldemar et al. (2019), Wallace et al. (2016), Zetterström et al. (2023)	“She's much more friendly. … I'm leading her places rather than she leading me” (Wallace et al. 2016, 1280)
Safety that opens the door to vulnerability	Feeling safe enough to be oneself (17 studies)	Bradley et al. (2021), Coelho et al. (2024), Eiroa‐Orosa and Incera‐Rosas (2025), Hammervold et al. (2022), Isobel (2019), Kehoe et al. (2023), Klevan et al. (2024), Lauzier‐Jobin and Houle (2024), Molin et al. (2018), Okumura and Katsuki (2024), Priebe et al. (2018), Prytz et al. (2019), Reed et al. (2018), Scheirich et al. (2024), Sellin et al. (2018), Vandewalle et al. (2019), Wallace et al. (2016)	“Trust, honesty, and respect from the nurse create a feeling of safety” (Priebe et al. 2018, 859) “Receiving care in the most peaceful seclusion room helped her to calm down…” (Hammervold et al. 2021, 478)
	From hesitation to openness (20 studies)	Banfield and Forbes (2018), Donaghay‐Spire et al. (2016), Eiroa‐Orosa and Incera‐Rosas (2025), Hristodoulidis et al. (2022), Karbouniaris et al. (2022), Keefe et al. (2020), Kehoe et al. (2023), Kirkegaard Thomsen et al. (2024), Klevan et al. (2024), Lauzier‐Jobin and Houle (2024), Molin et al. (2020), Priebe et al. (2018), Prytz et al. (2019), Reed et al. (2018), Reinius et al. (2023), Scheirich et al. (2024), Sellin et al. (2018), Twamley et al. (2021), Vandewalle et al. (2019), Walde et al. (2023)	“She made me feel comfortable … opened me up to being myself” (Kehoe et al. 2023, 5) “I think [Open Dialogue] kinda took that stigma away, that mental health isn't a bad thing” (Twamley et al. 2021, 497)
	Finding strength through expression (17 studies)	Donaghay‐Spire et al. (2016), Eiroa‐Orosa and Incera‐Rosas (2025), Faith et al. (2023), Hammervold et al. (2022), Kidd et al. (2015), Klevan et al. (2024), Lauzier‐Jobin and Houle (2024), Priebe et al. (2018), Reinius et al. (2023), Rooney et al. (2016), Scheirich et al. (2024), Twamley et al. (2021), Vandewalle et al. (2019), Walde et al. (2023), Waldemar et al. (2019), Wong et al. (2019), Zetterström et al. (2023)	“Verbalising the suffering becomes a way of understanding” (Priebe et al. 2018, 861) “Participants described how the Daily Talks intervention enabled them to access deep and heavy emotions better.” (Reinius et al. 2023, 764)
Encouragement that fuels forward movement	Encourage‐ment that lifts you (33 studies)	Banfield and Forbes (2018), Bradley et al. (2021), Donaghay‐Spire et al. (2016), Eiroa‐Orosa and Incera‐Rosas (2025), Faith et al. (2023), Horgan et al. (2021), Howell et al. (2023), Hristodoulidis et al. (2022), Igarashi et al. (2024), Jørgensen et al. (2022), Karbouniaris et al. (2022), Keefe et al. (2020), Kehoe et al. (2023), Kidd et al. (2015), Kirkegaard Thomsen et al. (2024), Lauzier‐Jobin and Houle (2024), Molin et al. (2018), Molin et al. (2020), Okumura and Katsuki (2024), Priebe et al. (2018), Prytz et al. (2019), Raitakari et al. (2018), Reed et al. (2018), Reinius et al. (2023), Rooney et al. (2016), Scheirich et al. (2024), Sellin et al. (2018), Sellin et al. (2019), Shue et al. (2023), Solomon et al. (2021), Vandewalle et al. (2019), Wallace et al. (2016), Zetterström et al. (2023)	“You need that patience. … When you do see that change happen, it's so rewarding” (Faith et al. 2023, 2184) “She sometimes says: ‘You may consider me to be your mom.’ … She is like a source of inspiration to me” (Karbouniaris et al. 2022, 30)
	Feeling genuinely heard (38 studies)	Banfield and Forbes (2018), Bradley et al. (2021), Coelho et al. (2024), Donaghay‐Spire et al. (2016), Eiroa‐Orosa and Incera‐Rosas (2025), Forchuk et al. (2021), Hammervold et al. (2022), Horgan et al. (2021), Hristodoulidis et al. (2022), Hyde et al. (2015), Isobel (2019), Jørgensen et al. (2022), Karbouniaris et al. (2022), Keefe et al. (2020), Kehoe et al. (2023), Kidd et al. (2015), Klevan et al. (2024), Lauzier‐Jobin and Houle (2024), Molin et al. (2018), Molin et al. (2020), Okumura and Katsuki (2024), Pelto‐Piri et al. (2019), Pfeiffer et al. (2019), Priebe et al. (2018), Prytz et al. (2019), Raitakari et al. (2018), Reinius et al. (2023), Scheirich et al. (2024), Sellin et al. (2018), Sellin et al. (2019), Shue et al. (2023), Twamley et al. (2021), Vandewalle et al. (2019), Walde et al. (2023), Waldemar et al. (2019), Wallace et al. (2016), Wong et al. (2019), Zetterström et al. (2023)	“I just wish they would listen to me. … I don't feel like I'm getting any better” (Hyde et al. 2015, 69) “I feel quite well when speaking to the nurses, … because someone is actually listening to me” (Coelho et al. 2024, 6)
Longing for depth and disappointment in its absence	Emotional support that feels real (39 studies)	Banfield and Forbes (2018), Bradley et al. (2021), Coelho et al. (2024), Donaghay‐Spire et al. (2016), Eiroa‐Orosa and Incera‐Rosas (2025), Faith et al. (2023), Forchuk et al. (2021), Hammervold et al. (2022), Horgan et al. (2021), Howell et al. (2023), Hyde et al. (2015), Igarashi et al. (2024), Isobel (2019), Jørgensen et al. (2022), Karbouniaris et al. (2022), Keefe et al. (2020), Kehoe et al. (2023), Kidd et al. (2015), Klevan et al. (2024), Lauzier‐Jobin and Houle (2024), Molin et al. (2020), Okumura and Katsuki (2024), Pelto‐Piri et al. (2019), Pfeiffer et al. (2019), Priebe et al. (2018), Prytz et al. (2019), Reed et al. (2018), Reinius et al. (2023), Rooney et al. (2016), Scheirich et al. (2024), Sellin et al. (2018), Sellin et al. (2019), Selvin et al. (2021), Solomon et al. (2021), Vandewalle et al. (2019), Walde et al. (2023), Waldemar et al. (2019), Wallace et al. (2016), Zetterström et al. (2023)	“I consider her to be like a confidante” (Lauzier‐Jobin and Houle 2024, 73) “It's like therapy, to talk it away with somebody who understands” (Sellin et al. 2019, 2088) “They didn't push me … I just felt safe” (Keefe et al. 2020, 204)
	Wanting more than medication (39 studies)	Bradley et al. (2021), Carmel et al. (2018), Coelho et al. (2024), Donaghay‐Spire et al. (2016), Eiroa‐Orosa and Incera‐Rosas (2025), Faith et al. (2023), Forchuk et al. (2021), Hammervold et al. (2022), Horgan et al. (2021), Howell et al. (2023), Hristodoulidis et al. (2022), Igarashi et al. (2024), Isobel (2019), Jørgensen et al. (2022), Keefe et al. (2020), Kehoe et al. (2023), Kirkegaard Thomsen et al. (2024), Klevan et al. (2024), Lauzier‐Jobin and Houle (2024), Molin et al. (2018), Molin et al. (2020), Pelto‐Piri et al. (2019), Pfeiffer et al. (2019), Prytz et al. (2019), Raitakari et al. (2018), Reed et al. (2018), Reinius et al. (2023), Rooney et al. (2016), Scheirich et al. (2024), Sellin et al. (2018), Sellin et al. (2019), Selvin et al. (2021), Solomon et al. (2021), Twamley et al. (2021), Vandewalle et al. (2019), Waldemar et al. (2019), Walde et al. (2023), Wallace et al. (2016), Wong et al. (2019), Zetterström et al. (2023)	“There is nothing to get someone through a bad day … just up the medication” (Kehoe et al. 2023, 7) “I think it is important that nurses have an awareness of community services where people can be referred to because mental illness is a lifelong journey, so what happens in hospital, or what happens in the short term, that's not it, so we need long‐term plans…maybe visiting community groups” (Horgan et al. 2021, 142) “The peer support worker was able to provide information about strategies” (Walde et al. 2023, 6)
	When things don't feel right (19 studies)	Banfield and Forbes (2018), Carmel et al. (2018), Eiroa‐Orosa and Incera‐Rosas (2025), Forchuk et al. (2021), Hammervold et al. (2022), Horgan et al. (2021), Howell et al. (2023), Hyde et al. (2015), Igarashi et al. (2024), Isobel (2019), Kehoe et al. (2023), Kidd et al. (2015), Molin et al. (2018), Pelto‐Piri et al. (2019), Scheirich et al. (2024), Selvin et al. (2021), Twamley et al. (2021), Vandewalle et al. (2019), Waldemar et al. (2019)	“They don't want me to dwell … but they're making me dwell” (Hyde et al. 2015, 70) “when changes were made and not communicated to patients, it created an atmosphere of frustration on the unit” (Molin et al. 2018, 1704)

**TABLE 6:** Overview of barriers and facilitators to implementing recovery‐oriented conversations.

Theme	Descriptor (*n*)	Supporting studies	Illustrative quotations and evidence
Timing and clarity: Gateways or roadblocks?	Readiness, timing, and complexity (48 studies)	Banfield and Forbes (2018), Bradley et al. (2021), Carmel et al. (2018), Coelho et al. (2024), Donaghay‐Spire et al. (2016), Eiroa‐Orosa and Incera‐Rosas (2025), Faith et al. (2023), Forchuk et al. (2021), Hammervold et al. (2022), Horgan et al. (2021), Howell et al. (2023), Hristodoulidis et al. (2022), Hyde et al. (2015), Igarashi et al. (2024), Isobel (2019), Jørgensen et al. (2022), Karbouniaris et al. (2022), Keefe et al. (2020), Kehoe et al. (2023), Kidd et al. (2015), Kirkegaard Thomsen et al. (2024), Klevan et al. (2024), Lauzier‐Jobin and Houle (2024), Molin et al. (2020), Molin et al. (2018), Okumura and Katsuki (2024), Pelto‐Piri et al. (2019), Pfeiffer et al. (2019), Priebe et al. (2018), Prytz et al. (2019), Raitakari et al. (2018), Reed et al. (2018), Reinius et al. (2023), Rooney et al. (2016), Scheirich et al. (2024), Sellin et al. (2018), Sellin et al. (2019), Shue et al. (2023), Solomon et al. (2021), Twamley et al. (2021), van Lankeren et al. (2020), Vandewalle et al. (2019), Waldemar et al. (2019), Walde et al. (2023), Wallace et al. (2016), Whittle et al. (2024), Wong et al. (2019), Zetterström et al. (2023)	“Several patients considered the time point of the PIR [post‐incident review] as too early after the restraint event, consequently they were not mentally capable to reflect” (Hammervold et al. 2022, 478) “Things happen in people's lives. They lose their home, their income, or other things, which makes what we talked about yesterday invalid today. We have to make redefinitions all the time” (Reed et al. 2018, 817) “In contrast, … all referred to not being ‘ready’ to talk and think at the beginning of their admission” (Donaghay‐Spire et al. 2016, 475)
	Quality of communication and information flow (39 studies)	Banfield and Forbes (2018), Bradley et al. (2021), Carmel et al. (2018), Coelho et al. (2024), Donaghay‐Spire et al. (2016), Eiroa‐Orosa and Incera‐Rosas (2025), Faith et al. (2023), Forchuk et al. (2021), Hammervold et al. (2022), Horgan et al. (2021), Howell et al. (2023), Hyde et al. (2015), Igarashi et al. (2024), Isobel (2019), Jørgensen et al. (2022), Karbouniaris et al. (2022), Keefe et al. (2020), Kehoe et al. (2023), Kidd et al. (2015), Klevan et al. (2024), Molin et al. (2020), Molin et al. (2018), Okumura and Katsuki (2024), Pelto‐Piri et al. (2019), Pfeiffer et al. (2019), Priebe et al. (2018), Raitakari et al. (2018), Reed et al. (2018), Reinius et al. (2023), Scheirich et al. (2024), Selvin et al. (2021), van Lankeren et al. (2020), Walde et al. (2023), Wallace et al. (2016), Whittle et al. (2024), Wong et al. (2019)	“If a patient does not know in a clear way what is happening …, he will not be able to change anything” (Eiroa‐Orosa and Incera‐Rosas (2025), 768) “Let's talk about it together … sets a good frame of therapy that's again non‐hierarchical” (Faith et al. 2023, 2185) “With a new service or worker it's like starting again. No history was shared‐or if it was they haven't read it” (Banfield and Forbes 2018, 6)
Power and presence matter	Power balance and participation (21 studies)	Banfield and Forbes (2018), Bradley et al. (2021), Donaghay‐Spire et al. (2016), Eiroa‐Orosa and Incera‐Rosas (2025), Hammervold et al. (2022), Karbouniaris et al. (2022), Keefe et al. (2020), Kidd et al. (2015), Kirkegaard Thomsen et al. (2024), Lauzier‐Jobin and Houle (2024), Molin et al. (2020), Molin et al. (2018), Pfeiffer et al. (2019), Priebe et al. (2018), Prytz et al. (2019), Reed et al. (2018), Reinius et al. (2023), Rooney et al. (2016), Scheirich et al. (2024), Twamley et al. (2021), Waldemar et al. (2019)	“I am clear on the question ‘What is it that you want?’ and believe that they should be allowed to decide. ‘What do you wish for? On what issues can we agree, what do you want help on? … How can we reach these goals together although we have a starting point from which we completely disagree?’” (Prytz et al. 2019, 4) “Participants repeatedly brought up the importance of Daily Talks being a space controlled by the patients, including aspects of both time and content” (Reinius et al. 2023, 764)
	Relational and emotional climate (47 studies)	Banfield and Forbes (2018), Bradley et al. (2021), Coelho et al. (2024), Donaghay‐Spire et al. (2016), Eiroa‐Orosa and Incera‐Rosas (2025), Faith et al. (2023), Forchuk et al. (2021), Hammervold et al. (2022), Horgan et al. (2021), Howell et al. (2023), Hristodoulidis et al. (2022), Hyde et al. (2015), Igarashi et al. (2024), Isobel (2019), Jørgensen et al. (2022), Karbouniaris et al. (2022), Keefe et al. (2020), Kehoe et al. (2023), Kidd et al. (2015), Kirkegaard Thomsen et al. (2024), Klevan et al. (2024), Lauzier‐Jobin and Houle (2024), Molin et al. (2020), Molin et al. (2018), Okumura and Katsuki (2024), Pelto‐Piri et al. (2019), Pfeiffer et al. (2019), Priebe et al. (2018), Prytz et al. (2019), Raitakari et al. (2018), Reed et al. (2018), Reinius et al. (2023), Rooney et al. (2016), Scheirich et al. (2024), Sellin et al. (2018), Sellin et al. (2019), Selvin et al. (2021), Solomon et al. (2021), Twamley et al. (2021), van Lankeren et al. (2020), Vandewalle et al. (2019), Waldemar et al. (2019), Walde et al. (2023), Wallace et al. (2016), Whittle et al. (2024), Wong et al. (2019), Zetterström et al. (2023)	“You can feel that really soon if your doctor or your nurse think that you are a hopeless case, even though they don't really show it, you can feel it” (Horgan et al. 2021, 142)
Fundamental systems that fit	Practical and structural conditions (25 studies)	Banfield and Forbes (2018), Bradley et al. (2021), Donaghay‐Spire et al. (2016), Faith et al. (2023), Hammervold et al. (2022), Horgan et al. (2021), Hristodoulidis et al. (2022), Igarashi et al. (2024), Isobel (2019), Jørgensen et al. (2022), Karbouniaris et al. (2022), Keefe et al. (2020), Kehoe et al. (2023), Kidd et al. (2015), Klevan et al. (2024), Lauzier‐Jobin and Houle (2024), Molin et al. (2020), Molin et al. (2018), Priebe et al. (2018), Raitakari et al. (2018), Reed et al. (2018), Shue et al. (2023), Solomon et al. (2021), Whittle et al. (2024), Zetterström et al. (2023)	“Every single day we meet with each individual veteran. … We talk about really everything that they're bringing to the table that they want to work on. … So every day we try to adjust the [treatment] plan to what spot the veteran is in” (Shue et al. 2023, 287)
	Systemic and cultural alignment (19 studies)	Banfield and Forbes (2018), Carmel et al. (2018), Eiroa‐Orosa and Incera‐Rosas (2025), Faith et al. (2023), Forchuk et al. (2021), Horgan et al. (2021), Hyde et al. (2015), Isobel (2019), Jørgensen et al. (2022), Karbouniaris et al. (2022), Kidd et al. (2015), Lauzier‐Jobin and Houle (2024), Priebe et al. (2018), Reed et al. (2018), Vandewalle et al. (2019), Wallace et al. (2016), Whittle et al. (2024), Wong et al. (2019), Zetterström et al. (2023)	“When the person's version of their state of being is not aligned with the discourse of the medically dominant environment … feelings of invalidation and dismissal are prevalent” (Hyde et al. 2015, 70) “The system is so fixed in what it believes … you get this conflict” (Kidd et al. 2015, 42)

### 3.1 Study Characteristics

The integrated review included 49 studies published in 2015–2025 that had a total of more than 2120 participants: patients (*n =* 1481), MHPs (*n =* 268), and mixed groups (*n =* 372+). Nearly half of the studies (49%) focused solely on patients (20 qualitative, *n =* 510; 4 quantitative, *n =* 971), whereas 24% examined only MHPs (11 qualitative, *n =* 214; 1 mixed‐method, *n =* 268). The remaining 27% included both groups (10 qualitative, *n =* 203+; 3 mixed‐method, *n =* 169+). Studies were conducted across 17 countries, mostly in Sweden (i.e., 9 studies), Australia (i.e., 8 studies), and the United States (i.e., 7 studies). One study (Horgan et al. 2021) spanned six countries, as shown in Table S1. The data were primarily collected through interviews (i.e., 30 studies), focus groups (i.e., 9 studies), and surveys (i.e., 7 studies), along with other methods such as field notes, observations, written feedback, validated scales, clinical records, and real‐life conversations. In qualitative studies, thematic analysis was the most common analytical method (i.e., 28 studies), followed by content analysis (i.e., 12 studies), triangulation or member checking (i.e., 9 studies), grounded theory (i.e., 5 studies), and phenomenological or interpretative approaches (i.e., 8 studies). Beyond that, six studies employed quantitative methods, including descriptive and regression analysis (see Table S1).

### 3.2 Conceptualising ROCs

The integrated review revealed a rich, multifaceted understanding of ROCs in psychiatric care. As outlined in Table 2, three interconnected conceptual strands were identified: relational practices sustained through everyday contact, shared spaces for structuring talk and direction, and conversations grounded in personal and social realities.

Across the studies, the dominant narrative suggested that ROCs are first understood as relational practices sustained through everyday contact. Instead of being confined to formal therapeutic settings, they seem to occur organically in routine as well as supportive and humanising interactions. That trend was apparent in 28 studies, in which ROCs were described as relational anchoring practices rooted in trust and shared presence. Nearly all the studies (i.e., 48 studies) understood ROCs as a supportive presence that underscores shared humanity and emotional sensitivity. A subset of 12 studies further illuminated how rhythmic engagement in silence, humour, or the intonation of speech functions as a subtle yet potent channel for affirming personhood and deepening relational ties. In 19 studies, those emotion‐focused interactions were typically characterised by respectful communication marked by empathy and a sense of presence.

A second conceptual strand of studies conceived ROCs as shared spaces for structuring talk and direction. Therein, the emphasis shifted to understanding ROCs as a mechanism for cultivating agency, sharpening clarity, and enabling shared decision‐making. In 18 studies, ROCs were described as voice‐centred exchanges that prioritised attentive listening and gave a genuine lift to patients' perspectives. In 15 studies, as clarity‐oriented communication was reinforced, ROCs used easy‐to‐understand language that fostered shared understanding. Beyond that, 10 studies underscoring the importance of choice‐driven dialogue highlighted how ROCs were viewed as restoring a sense of agency and empowering individuals to shape their own trajectories of recovery. That conceptual framing casts ROCs as instruments that enable patients to navigate complex care systems as active participants instead of passive recipients.

A third and equally vital narrative depicted ROCs as conversations grounded in personal and social realities. Instead of being abstract or detached, those exchanges were saturated with people's lived experiences, cultural identities, and the surrounding social environment. In eight studies, ROCs were characterised as context‐responsive talks that could be adapted to each individual's circumstances. Moreover, when conversations were tied to narrative, they were described as story‐linked dialogues that served as a powerful means for people to interpret their experiences with mental health. Those narrative arenas opened a path to examine identity, wrestle with meaning, and envision possibilities, all while challenging narrow storylines and reinforcing personal agency.

### 3.3 Facilitating ROCs in Practice

ROCs in care were shaped by the formation of conversational approaches that fostered therapeutic relationships. Those themes were presented in four areas: deepening dialogue through emotional and narrative work, dialogically co‐constructing recovery‐oriented pathways, being present to support conversation, and designing conditions that invite conversation (see Table 3).

#### 3.3.1 Deepening Dialogue Through Emotional and Narrative Work

A striking strand was the use of narrative techniques to deepen therapeutic conversations. In 44 studies, reflexive and meaning‐making dialogues were catalysts for recovery. Those exchanges facilitated co‐reflection and narrative reconstruction that enabled patients to make sense of their experiences and envision new possibilities. In 13 studies, MHPs supported such emotional expression by validating patients' feelings, which offered them a hopeful outlook and nurtured a safe, non‐judgemental atmosphere. Such actions also strengthened the therapeutic alliance, thereby underscoring narrative's deep transformative power in recovery‐oriented care.

#### 3.3.2 Dialogically Co‐Constructing Recovery‐Oriented Pathways

A second recurring strand in the studies was the co‐creation of recovery journeys via mutual dialogues between patients and MHPs. In 20 studies, instead of imposing a fixed blueprint for treatment, practitioners shifted towards collaborative planning and decision‐making. That approach was operationalised by being involved in care plans, negotiating strategies, engaging in daily routines, and charting future goals. In six studies, the use of tools and structured aids, including SHARE sheets, visual maps, and goal‐setting templates, further helped to initiate and sustain dialogue, which provided continuity and reference points. Inclusive and equal participation, as described in five studies, was enhanced by meeting formats, user‐led discussions, and built‐in mechanisms that ensured the equal weight of every voice. That strategy ended up being a game‐changer that widened the space and anchored the recovery process in a more authentic tone, especially when integrated with peer support and lived experience in three studies. Supporting those strands, quantitative data showed that collaborative methods were linked to reduced conflict and increased opportunities for user‐led dialogue (Banfield and Forbes 2018; Igarashi et al. 2024; Okumura and Katsuki 2024).

#### 3.3.3 Being Present to Support Conversation

Third, the studies repeatedly emphasised that relational presence and emotional sensitivity were crucial for sustaining ROCs. In 18 studies, MHPs who utilised supportive communication techniques—for example, deep listening, gentle pacing and a calm tone, and displays of genuine empathy—were consistently described as being more effective at fostering trust and openness. In three studies, informal and everyday talk, whether over a steaming mug of coffee or a brief catch‐up about daily minutiae, acted as the glue that builds rapport and narrows the emotional distance between patients and MHPs. In five studies, such relational grounding helped MHPs to shift in collaborative ways that encouraged social reconnection and fostered equality. Doing so cultivated therapeutic relationships, as observed in 10 studies, that were based on reciprocal honesty, steady consistency, and shared reflection.

#### 3.3.4 Designing Conditions That Invite Conversation

In time, the structural and environmental context in which ROCs took place proved to be a significant factor that shaped both the depth and impact of the conversations. As described in 13 studies, participants repeatedly emphasised that the environment supported communication. Those environments included calm, familiar spaces such as kitchens, gardens, and other homey settings and featured flowers, food, and/or music, all as elements that encouraged warm, spontaneous conversations that reduced tension. In three studies, cultural and contextual sensitivity further encouraged the inclusivity and relevance of those conversations. Gender‐responsive environments, interpreters, and culturally grounded practices were among the strategies that supported patients' diverse needs. In four studies, arenas for recovery‐oriented group dialogue, using methodical formats and interweaving narratives, were described as fertile terrain for cultivating relational bonds and co‐creating shared meaning.

### 3.4 Alignment of ROCs With the CHIME Framework

In focusing on connectedness, hope, identity, meaning, and empowerment, ROCs aligned with the CHIME framework and thus nurtured relationships that validated individuals' sense of self and encouraged trajectories of recovery. Each CHIME domain was deductively examined and interpreted according to recurring patterns and its theoretically grounded indicators (see Table 4).

#### 3.4.1 Connectedness

ROCs consistently nurtured a sense of connectedness by shaping environments that fostered relational engagement and interpersonal presence. In 29 studies, participants reported that support from others, especially MHPs, was felt through listening, emotional availability, and a steady, reliable presence. Such exchanges often became anchor points of stability and care. Trusting relationships represented another key facet of connectedness, with 22 studies highlighting how ROCs nurtured intimacy and reciprocal ties with staff and peers. Those bonds, extending beyond the clinical sphere, delivered a sense of mutual respect and shared humanity. In eight studies, community involvement was observed in shared spaces, particularly in the flow of social activities, which enabled individuals to reconnect with one another and re‐enter communal life. ROCs frequently acted as bridges that opened pathways to broader social participation and cemented a sense of belonging. Furthermore, peer support and group‐based conversations, as reported in eight studies, provided opportunities for mutual understanding and genuine solidarity. What mattered most to participants was the tapestry of shared moments and the emotional echoes offered by peer‐run spaces, which mitigated loneliness and wove a strand of recovery among patients.

#### 3.4.2 Hope

Hope, viewed as a cornerstone of ROCs, wove its way through conversations that fostered confidence in recovery and envisioned what might lie ahead. In 25 studies, a belief in recovery, with MHPs reinforcing confidence by radiating optimism, affirmed recovery‐focused goals and encouraged individuals to envision a future for themselves. In 14 studies, ROCs also helped to fuel motivation for change, whereas goal‐setting and forward‐looking conversations encouraged individuals to take steps towards personal growth. Those dialogues were often described as sparks that energised people to pursue transformation. Moreover, positive thinking and optimism, as described in 20 studies, were nurtured by reframing challenges and drawing attention to emotionally uplifting exchanges and personal strengths. Last, in six studies, ROCs became vehicles for valuing success and achievement as colleagues and experts marked key milestones and validated forward movement. Those acknowledgements were described as reinforcing hope and serving as psychological and relational anchors.

#### 3.4.3 Identity

ROCs provided a space where patients could explore, validate, and reshape their identities beyond the shadow of illness. In 28 studies, rebuilding a positive sense of self flourished when dialogue spotlighted each person's singular sense of agency and inherent dignity. Those dialogues enabled patients to reclaim narratives that diagnostic labels had overshadowed. In 20 studies, ROCs reflected self‐acceptance, thereby revealing how ROCs can affirm personal reflections and nurture identity as a lived, ever‐changing narrative. The journey often required acknowledging both vulnerability and strength. In another 14 studies, ROCs helped with overcoming stigma, especially by confronting diagnostic language and reframing identity through more humanising dialogue. Such exchanges empowered individuals to resist internalised stigma and claim their personhood. Last, personal growth, as evident in 14 studies, was embodied in ROCs that fostered autonomy, transformation, and active engagement in recovery. Those interactions encouraged developing identity as an empowering process.

#### 3.4.4 Meaning

ROCs have proven to be essential in helping people to find renewed meaning and purpose in the face of illness. In 18 studies, ROCs reflected meaningful life roles by engaging individuals in dialogues that promoted autonomy, set clear goals, and encouraged a return to valued pursuits, including work and volunteering. In 30 studies, ROCs reflected social goals and responsibilities via collaborative planning, peer involvement, and culturally sensitive dialogues that resonated with each person's values and social context. In 27 studies, quality of life was enhanced through conversations that validated emotions, supported regulation, encouraged self‐expression, and promoted meaningful engagement. Moreover, in six studies, ROCs carved out a niche for spirituality and existential reflection, which allowed individuals to explore the weighty questions of meaning, purpose, and identity, often characterised as grounding and transformative.

#### 3.4.5 Empowerment

Empowerment was observed in heightened agency, expanded autonomy, and increased self‐efficacy. The prominent indicator of control over one's life was reflected in 44 studies. ROCs underpinned shared decision‐making, encouraged self‐determination, and allowed people to shape their own journeys of recovery, which were associated with trust and deeper engagement. In 10 studies, personal responsibility was nurtured through conversations that encouraged individuals to voice their needs, weigh options, and take ownership of their recoveries. In 15 studies, ROCs focusing on strengths reinforced individuals' capabilities and nurtured confidence in their ability to cope and thrive. In 14 studies, ROCs also encouraged self‐management through reflective dialogue and using tools that nurtured independent coping and emotional regulation.

### 3.5 Experiences and Perceived Outcomes of ROCs

ROCs were viewed as deeply humanising moments in psychiatric care that carved out spaces where emotions could be voiced, understanding could be shared, and personal growth could occur. As Table 5 shows, those trends contributed to four themes: safety that opens the door to vulnerability, longing for depth and disappointment in its absence, encouragement that fuels forward movement, and feeling human in the eyes of others.

#### 3.5.1 Safety That Opens the Door to Vulnerability

A recurring theme in the studies was the transformative role of psychological safety in facilitating openness. For patients in 20 studies, ROCs began with a palpable feeling of hesitation that led to openness. Some felt that they had embarked on a journey that was unsafe, driven by worry due to past experiences of coercion, mistrust, and/or the persistent dread of being judged. Meanwhile, others initially felt guarded or sceptical but gradually shifted towards openness. However, in 17 studies, when patients encountered MHPs who conveyed warmth and maintained a steady, attentive presence, they felt safe enough to be themselves. The notion of safety extended beyond the mere absence of harm by resonating with the rhythm of attentive hearing and secure emotional containment. In another 17 studies, many participants reported finding strength through expression when reflecting on the emotional release experienced while speaking uninterrupted. The fact that MHPs could refrain from their instinctive desire to fix things immediately allowed patients to process trauma and reclaim their agency. One participant in Hammervold et al. (2022, 477) reflected that the liberty to speak without restraint “felt good”, which helped with transcending the pain of coercive experiences. Quantitative data reinforced those narratives by linking safety to transparent decision‐making, deeper trust, greater clarity, and more genuine therapeutic relationships (Okumura and Katsuki 2024; Priebe et al. 2018; Wong et al. 2019). However, not all experiences were positive; some participants described feeling overwhelmed or exposed, thereby underscoring that safety is a negotiated process.

#### 3.5.2 Encouragement That Fuels Forward Movement

In 38 studies, feeling genuinely heard was the dominant descriptor, whereas encouragement that lifts people, observed in 33 studies, ignited a strong drive for change among patients and MHPs. Patients repeatedly felt that ROCs brought them relief, a sense of validation, and a rekindled sense of connection. Such feelings often blossomed into motivation and renewed hope, especially when conversations turned towards personal goals and the possibilities that lie ahead. The emotional resonance of those moments felt almost tangible. As one participant in Coelho et al. (2024, 6) put it, “I feel quite well when speaking to the nurses. … I feel relieved because someone is actually listening to me”. The MHPs echoed that sentiment by describing a deep sense of fulfilment that surfaced while witnessing change take shape. As an MHP in Faith et al. (2023, 2185) stated, “When you … see that change happen, it's so rewarding”. Quantitative data also supported those lived accounts by tying nurturing dialogue to reduced uncertainty, increased confidence, and a firmer sense of progress (Howell et al. 2023; Okumura and Katsuki 2024; Wong et al. 2019). By contrast, when encouragement fell short, participants described feeling as though they were being brushed aside or hurried, thereby highlighting the motivational power of encouragement in ROCs and its role in maintaining momentum towards recovery.

#### 3.5.3 Longing for Depth and Disappointment in Its Absence

Although many participants praised ROCs for their richness and relational weight, a notable subset voiced a yearning for deeper, more substantial engagement. In 39 studies, both patients and MHPs emphasised the importance of receiving or offering emotional support that felt real. For some, ROCs resembled therapy by affording a space to discuss their distress with someone who understood, which prompted greater trust, deeper insight, and a renewed sense of hope. In 39 studies, participants found themselves wanting to give or receive more than medication, and when discussions remained shallow or revolved exclusively around medication, patients described feeling frustrated, emotionally detached, or misunderstood. Similarly, MHPs also observed that a conversation rich in meaning often produced an effect that outstripped the impact of clinical interventions applied in isolation. When the hoped‐for depth fell short, disappointment crept in. In 19 studies, participants recounted episodes where dialogue felt wrong or purely transactional, which triggered disengagement and a lingering sense of being overlooked.

#### 3.5.4 Feeling Human in the Eyes of Others

Another profound aspect of ROCs was their being experienced as affirmations of personhood. In 28 studies, patients reported being treated as people because they felt seen—not merely as patients or diagnostic labels but as unique, complex individuals. Many described that recognition as an experience that helped to rebuild self‐worth and steel themselves against the dehumanising side effects of psychiatric treatment. As a participant in Eiroa‐Orosa and Incera‐Rosas (2025) summed it up, “We all start from the point: being people” (p. 771). In 19 studies, those moments of recognition felt emotionally grounding and reinforced the therapeutic alliance, thereby ensuring a connection that felt mutual among patients. Similarly, MHPs described ROCs as opportunities to connect on a human level and nurture dignity and respect. Quantitative findings further linked emotional validation and perceived respect from MHPs to improved outcomes of recovery (Okumura and Katsuki 2024; Scheirich et al. 2024; Wong et al. 2019). However, when that acknowledgement was absent, participants reported feeling ignored, mislabelled, or reduced to nothing more than their symptoms. Those divergent accounts highlight the dual capacity of ROCs to either affirm or erode personhood depending on the quality of engagement.

### 3.6 Implementing ROCs in Psychiatric Care

Implementing ROCs in psychiatric settings was a complex, context‐sensitive process. Across the studies, three intertwined subgroups were identified: timing and clarity as gateways or roadblocks; power and presence matter; and fundamental systems that fit (see Table 6).

#### 3.6.1 Timing and Clarity: Gateways or Roadblocks?

Nearly all the studies (i.e., 48 studies) showed that readiness, timing, and acknowledging individual complexities were paramount to the effectiveness of ROCs. Both patients and MHPs stressed that ROCs depended on the individual's readiness and the appropriateness of the moment. Conversations initiated during acute crises were overwhelming and disengaging, and participants described them as burdensome or poorly timed. By contrast, when ROCs were introduced when patients felt emotionally prepared, they tended to be experienced as genuinely meaningful and supportive. Moreover, in 39 studies, the quality of communication and flow of information were not fixed, for the impact shifted according to how they occurred and the depth to which they probed. Some patients found frequent but brief informal check‐ins to be helpful, whereas others preferred fewer but more in‐depth conversations. Communication was thus a make‐or‐break activity, for vague, poorly structured conversations eroded trust, whereas co‐created agendas, clear goals, and a systematic follow‐up routine encouraged engagement and continuity. When a holistic perspective was taken that recognised individuals instead of only their diagnoses, it tended to strengthen therapeutic rapport and enhance the perceived value of ROCs.

#### 3.6.2 Power, Presence, and the Relational Climate

The interplay between MHPs and patients played a pivotal role in shaping the implementation and impact of ROCs. Forty‐seven studies emphasised the crucial need to cultivate a relational and emotional climate where MHPs would feel bold enough to engage in authentic conversations. Likewise, the quality of ROCs consistently outweighed their quantity. Thoughtful, attuned dialogues tended to foster trust and recovery, whereas frequent but shallow exchanges could paradoxically tighten the knot of disengagement. Staff who flexibly responded to each person's needs and stayed emotionally aligned were better at nurturing conversations that felt genuine and trustworthy. Moreover, in 21 studies, MHPs' willingness and ability to balance power between themselves and patients, while encouraging them to participate in their care, formed the backbone of meaningful engagement. When they began with empathy, respect, and a flexible stance, patients were more likely to feel heard and valued. Nevertheless, systemic constraints often undermined those intentions. Rigid routines, administrative burdens, and staff shortages led to rushed, checklist‐like interactions that felt superficial and/or insincere. In such contexts, MHPs reported feeling overwhelmed, and some resisted adopting ROCs when they challenged traditional hierarchies or clinical norms.

#### 3.6.3 Fundamental Systems That Fit

Beyond individual interactions, the broader organisational and systemic context significantly influenced the sustainability of ROCs. In 25 studies, practical and structural conditions, including staffing levels, leadership support, and the prevailing institutional culture, emerged as key factors shaping the consistency and depth of the practical implementation of ROCs. In systems dominated by risk management and standardisation, patients often felt sidelined, and ROCs risked becoming procedural instead of staying rooted in person‐centred care. High staff turnover, limited resources, and persistent stigma, particularly towards individuals with complex needs, further constrained the potential of ROCs. By contrast, in 19 studies, when systemic, culturally responsive approaches were taken, they were far more likely to nurture implementation that felt meaningful and durable. Modest everyday actions, including daily check‐ins, peer involvement, and multidisciplinary teamwork, contributed significantly to embedding ROCs into the fabric of routine care. Strong leadership, continuous training, and the integration of perspectives emphasising lived experience proved to be essential to cultivating a conversational culture that improved recovery. When an organisation prioritised its recovery‐oriented values, employees felt driven, and patients appreciated conversations that were constant, inclusive, and genuinely empowering.

## 4 Discussion

In our integrative review, we aimed to synthesise diverse evidence on how ROCs are understood, practised, and experienced and their influencing factors in psychiatric care settings. On the basis of a narrative synthesis of 49 empirical studies across 17 countries, the findings suggest that ROCs are not merely an exchange of words but deeply relational, context‐aware, and potentially transformative practices that align with the CHIME framework, as outlined in Tables 2, 3, 4, 5, 6.

This review highlights a developing understanding of ROCs as relational practices embedded in everyday life, as structured dialogues that foster agency and clarity, and as contextually grounded conversations rooted in personal and social realities. Collectively, those findings suggest that ROCs are not limited to therapeutic sessions but may instead unfold in ordinary interactions shaped by mutual recognition and meaning‐making across diverse psychiatric settings. As mentioned in the introduction, Andvig and Biong's study (Andvig and Biong 2014) supports that understanding of ROCs by illustrating the importance of relational depth and the potential role of emotional presence within therapeutic settings. Similarly, McAllister et al. (2019) have described nurse–patient engagement as a co‐crafted process rooted in a shared understanding that unfolds over time. The spotlight on communication, emotional resonance, and storytelling in this review appears to signal a shift towards humanising psychiatric care, a strand observed by De Ruysscher et al. (2020), who have noted that inpatient recovery‐oriented practices can help to nurture dignity and forge emotional connections. Kornhaber et al. (2016) have further highlighted the essence of building therapeutic relationships, which may require a sustained emotional investment that can prove to be challenging in under‐resourced settings. Moreover, foundational work by Anthony (1993) and Davidson et al. (2005) has framed recovery as a relational journey, thereby laying the theoretical groundwork for viewing ROCs as humanising, co‐created engagements.

This review suggests that patients and MHPs consistently portray ROCs as resonant and humanising but nevertheless express mixed feelings about their potential capacity to recover and the disappointment that may follow when interactions feel shallow. Some participants described the process as superficial or hurried, a critique that seems to echo the findings of Hammervold et al. (2019), whose analysis has highlighted that conversational approaches, including post‐incident reviews, can marginalise that aspect and thus hinder their ability to promote recovery. Those experiences reflect broader concerns that, despite being widely endorsed, principles of recovery may sometimes be more rhetorical than practical because of limited relational engagement, organisational support, and systemic barriers (Karadzhov 2023; Melillo et al. 2025; Nakanishi et al. 2021). However, Ballard et al. (2024) have reiterated that communication practices that convey cues such as presence, attentiveness, and kindness while offering space tend to nurture deep dialogic connections, thereby supporting recovery in healthcare settings.

Our review also indicates how ROCs align with the CHIME framework in practice. It suggests that ROCs nurture connectedness by strengthening relationships and fostering community, while also sowing hope through the affirmation of goals. Moreover, ROCs encourage identity by confronting stigma, crafting meaning via dialogue, and encouraging empowerment by supporting autonomy. That practical mapping of ROCs appears to shift CHIME from a framework into a more usable clinical guide that offers MHPs actionable insights in psychiatric settings. Hare‐Duke et al. (2023) have suggested in their review that, although CHIME is important in shaping recovery, it needs refinement to ensure responsiveness in various contexts. Indeed, Jagfeld et al. (2021) had already broadened that perspective by applying the CHIME framework to include individuals with mental health disorders and thereby emphasising that recovery models need to remain adaptable and be capable of handling the complex intricacies of mental health conditions. This review suggests that ROCs organically embody CHIME's principles in everyday therapeutic exchanges, which reinforces findings from earlier studies. Leamy et al. (2023) have strengthened CHIME's practical relevance by surveying the instruments used to evaluate the orientation towards recovery, thus illustrating that the framework can serve both as a guiding concept and as a concrete, measurable benchmark for personal recovery.

However, Chatwiriyaphong et al.'s (2025) recent synthesis examining Southeast Asian cultural contexts appears to challenge the universality of the CHIME framework. Those authors note that recovery often incorporates collective values, familial interdependence, and a reliance on spiritual beliefs, which the original CHIME formulation (Leamy et al. 2011) does not fully capture. Similarly, in their review of recovery guidelines, Subandi et al. (2023) have expanded that cross‐cultural perspective by highlighting that such frameworks may need to be negotiated under suitable conditions and with careful attention to each setting's cultural, spiritual, and systemic nuances, as well as its capacity to respond. Consequently, when MHPs draw on ROC‐focused approaches, the resulting recovery‐oriented practices should remain adaptable and culturally sensitive while steering clear of ideas about what recovery means.

Our review also suggests four potential strategies for implementing ROCs: collaborative conversation, emotional presence, deep and meaningful dialogue, and supportive environments. When viewed as a whole, the evidence suggests leaning towards methods that prioritise care, thereby allowing patients to move beyond the role of passive recipients and become active participants in shaping their own narratives of recovery. An earlier synthesis by Hartley et al. (2020) has suggested that such relational interventions can strengthen the bond between nurses and patients and thereby improve outcomes of recovery. Meanwhile, Beyene et al. (2024) have described relational competence as a blend of empathy, emotional regulation, and authentic presence. In our review, casual exchanges, including off‐the‐cuff conversations and brief moments of connection, also emerged as being as impactful as formal interactions. By weaving those strands together, ROCs may transcend simple dialogue to become potent relationally transformative interventions. A previous study by Macdonald (2016) on the importance of small talk in nurse–patient interaction has affirmed that perspective by reframing everyday clinical dialogue as a form of professional competence that may help to ease patients into procedures, manage their rhythm of interaction, and foster relational trust. Newell and Jordan (2015) have further supported that notion in their own review by highlighting the importance of nurse–patient interaction for patients' experiences and recovery. At the same time, Hartley et al. (2020) have cautioned that those emotional connections often require structural, intentional effort to achieve emotional depth, thereby highlighting the need for conversational training and reflective practice when implementing ROCs.

Embedding ROCs in psychiatric practice also appears to demand more than a few procedural tweaks. On the contrary, it calls for a cultural revamping of the organisation's values and systems. Instead of treating the rollout of ROCs as a simple task, this review reframes it as a potential cultural shift that hinges on aligning the organisation with recovery‐focused values, securing leadership backing, and cultivating truly inclusive settings. This review highlights key factors that have shaped the implementation of ROCs, namely timing, staff and patient readiness, the quality of communication, and leadership support. As Lorien et al. (2020) have indicated, when an organisation cultivates a culture and couples it with decisive leadership, recovery‐oriented practice is far more likely to thrive. Furthermore, per Ashcraft et al. (2024), the rollout of practices cannot be one‐size‐fits‐all; instead, approaches to implementation have to be flexibly adapted to the array of healthcare environments. Such a technical, managerial lens seems to somewhat contrast the relational emphasis of ROCs, thereby sparking a lingering doubt about whether the two can be reconciled across differing logics of implementation. Moreover, systemic support for MHPs is needed to attain such relational competence (Jørgensen and Rendtorff 2018). That sentiment similarly resurfaced in Jørgensen et al. (2024), who have reported that both MHPs and patients appreciate dialogue, even as staff contend with obstacles such as time pressure and insufficient training. Abraham et al. (2024) have also identified obstacles in African contexts, including hierarchical staff–patient dynamics and limited training, thereby underscoring the significance of culturally responsive adaptations. Similarly, in Sweden, peer support workers have reported feeling marginalised due to unclear expectations and experiencing stigma from the MHPs, which highlights that though peer involvement may be embraced, institutional culture may nevertheless hinder their integration into practice (Wall et al. 2022).

### 4.1 Strengths and Limitations

This review offers methodological strengths by compiling evidence from 49 studies using Whittemore and Knafl's (2005) integrative systematic review design. The approach helped to maintain a rigorous process, thereby enabling a deep understanding of ROCs. Moreover, combining deductive coding under the CHIME framework and the inductive approach helped to cultivate and guide the nuanced development of our synthesis. Although the CHIME framework provided a scaffold, it may have also constrained the search for alternative paradigms of recovery. Using an inductive lens in the other five RQs may have helped to capture those overlooked nuances. Furthermore, the involvement of SURG helped to integrate lived‐experience perspectives, which were positioned at the core of the analysis; however, doing so may have introduced a degree of interpretive subjectivity that should be acknowledged. This review has shown transparency in its preregistration in PROSPERO, its adherence to PRISMA guidelines (Page et al. 2021), and in providing transparent reporting using the PCC framework (Peters et al. 2015).

Some studies published in journals or by publishers were criticised for inconsistent peer review, which may have affected the robustness of the evidence. Nevertheless, all studies underwent a transparent quality appraisal using the MMAT (Hong et al. 2018), enabling readers to assess credibility and transferability. Variation in methods and study quality may also have influenced the synthesis. Although a single reviewer did data extraction and analysis, interpretations were discussed within the research group, which may have reduced bias. A meta‐analysis was not feasible, for qualitative studies cannot be statistically pooled, and quantitative studies were too heterogeneous in design and outcomes to allow meaningful statistical synthesis. The patchy coverage across groups, coupled with a heavy reliance on participants' own reports, may further narrow the generalisability of the results. Excluding studies not written in English, though necessary for understanding, may have sidelined research with cultural resonance.

## 5 Conclusion

This integrated review suggests that person‐centred practices such as ROCs represent a cornerstone for personal recovery in psychiatric care. By cultivating safety, instilling hope, co‐creating meaning, and nurturing therapeutic bonds, ROCs offer mental health nurses a way to engage patients beyond the routine of clinical tasks. Altogether, the evidence narrows the field's focus and reaffirms that neither quality nor authenticity should be compromised in ROCs. Nevertheless, despite their promise, a web of systemic, cultural, and organisational hurdles still appears to hinder full integration in practice. Future research in various psychiatric settings should therefore investigate the long‐term effects of ROCs on recovery outcomes and assess their adaptability to diverse clinical environments. With the clear call for culturally responsive ROCs and the need for training among staff, future research should be tailored to pinpoint those gaps among mental health nurses and prioritise practical training to ensure that ROCs are adequately implemented into practice. Including co‐produced training packages with nurses and patients from diverse cultural and clinical backgrounds could be crucial to ensuring that the developed packages align with real‐world needs.

### 5.1 Relevance to Clinical Practice

This integrative review provides mental health nurses with tools to integrate ROCs into their everyday practice. ROCs may act as a safety net for emotions, add layers to patients' stories, and accommodate joint decision‐making, thereby strengthening therapeutic alliances and facilitating personal recovery. The findings indicate three pillars that nursing should reinforce: clinical competence, cultural sensitivity, and robust organisational scaffolding. Looking ahead, researchers should explore how ROCs can be reshaped and sustained while also addressing the need for rigorous quantitative research to examine their effects, including comparisons between compulsory and voluntary care, which stands to strengthen the evidence base for future practice.

## Author Contributions

Promise Ezinne Ekezie led the design and coordination of the review and took primary responsibility for drafting the manuscript. Alexander Rozental contributed to the title, abstract, and full‐text screening and supported the critical appraisal process. Git‐Marie Ejneborn‐Looi assisted with the title and abstract screening and offered valuable input during data interpretation. Ursula Werneke provided conceptual and methodological guidance, which helped to ensure theoretical coherence and relevance to psychiatric care. Sebastian Gabrielsson, as a principal supervisor, oversaw all phases of the review, contributed to the screening and appraisal processes, and offered overall academic and practical support throughout the project. In addition, Alexander Rozental, Git‐Marie Ejneborn‐Looi and Ursula Werneke acted as co‐supervisors to Promise Ezinne Ekezie by providing ongoing conceptual and theoretical insight, methodological feedback that strengthened the synthesis, and thoughtful reflections that enhanced the interpretive depth of the review, together with Sebastian Gabrielsson. All authors read and approved the final version of the manuscript.

## Funding

The work was funded by the Swedish Research Council (Grant: 2023‐06474_VR); however, the funders had no role in the study design, data collection, analysis, interpretation, or writing of the manuscript.

## Conflicts of Interest

Ursula Werneke has received lecture honoraria from Lundbeck and Janssen and has served on scientific committees for Jansen and Teva, which have also conferred honoraria to her. All other authors have no conflicts to declare.

## Supporting information


**Table S1:** Detailed characteristics of included studies.

**Table S2:** Quality appraisal of included articles using the Mixed Methods Appraisal Tool (MMAT).

## Data Availability

The data that support the findings of this study are available from the corresponding author upon reasonable request.
