# Supplementary material for: Recovery‐Oriented Conversations in Psychiatric Care: An Integrated Systematic Review
Source: Int J Ment Health Nurs. 2026 Jun 22;35(3):e70295. doi: 10.1111/inm.70295 (PMC13284807; doi:10.1111/inm.70295)
Supplement: Supplementary file 1 — Table S1: Detailed characteristics of included studies. [file INM-35-0-s001.docx]

### **Supplementary Table 1.1**

**Detailed characteristics of included studies.**

| Author(s), Year, Country | Aim | Recruitment method and channel | Population (P) | Concept (C) | Context (C) | Data collection method | Analytical method | Key findings | MMAT overall appraisal outcome |
| --- | --- | --- | --- | --- | --- | --- | --- | --- | --- |
| Qualitative Studies | | | | | | | | | |
| Bradley et al. (2021),  Australia | To gain an understanding of the acute mental health inpatient experience as described by Aboriginal women during admission | *Method:* Convenience sampling; purposive sampling  *Channel:* Clinic patients and users; direct and indirect contact via networks, organisations, and/or gatekeepers | 16 participants: 11 Aboriginal women, admitted to the inpatient unit, and 5 Aboriginal reference group members with experience in the inpatient unit as clinicians, carers, or mental health professionals (MHPs) | Exploring Aboriginal women’s experiences in acute mental health care by focusing on cultural safety, self-determination, (dis)connection, coercion, and communication while highlighting narratives of kinship, land, identity, and culturally responsive care strategies | Acute mental health inpatient unit in the Top End of the Northern Territory that serves clients from remote and urban areas of Australia | Field notes, individual interviews | Thematic analysis | The study showed that formal care lacked effective communication but that informal, culturally grounded dialogue, especially with Aboriginal mental health workers, fostered connections, identity, and empowerment. Key enablers included yarning, peer support, kinship, and cultural safety. | The study was methodologically rigorous and culturally sensitive, which aligned with its aims of inductive analysis and engagement with Aboriginal communities. Despite limitations, including restricted interpreter access and convenience sampling, the study maintained qualitative integrity through an ethical design and transparent reporting. |
| Carmel et al. (2017), US | To identify self-reported interpersonal barriers to recovery in individuals with borderline personality disorder (BPD) by exploring how family, friends, and providers may reinforce problematic behaviours that interfere with recovery goals | *Method:* Convenience sampling  *Channel:* Clinic patients and users | 31 patients who were psychiatrically disabled or unable to work for at least 6 months | Examining interpersonal barriers to BPD per the dialectical biosocial model recovery while highlighting dysfunctional behaviours, stigma, and low expectations and emphasising person–environment dynamics | Outpatient psychiatric clinic (i.e. Harborview Mental Health, Seattle, WA) US) involving participants in dialectical behaviour therapy (DBT) or applying for DBT-ACES, a programme promoting employment and self-sufficiency | Written feedback | Content analysis, grounded theory | The study showed that recovery conversations were often undermined by communication reinforcing dependency and low expectations. Instead of fostering empowerment, interactions validated avoidance and highlighted the need for intentional, strengths-based communication aligned with principles of recovery. | The study was methodologically robust qualitative research with clearly grounded findings, but the absence of reported ethical approval and consent procedures weakened it. |
| Coelho et al. (2024),  Portugal | To explore the perspectives of adult patients with mental health disorders on their relationship with nurses—its importance, their expectations, and the attitudes and skills that they expect from nurses | *Method:* Purposive sampling  *Channel:* Direct and indirect contact via networks, organisations, and/or gatekeepers; patient and user organisations | 8 patients | Therapeutic nurse–patient relationships from the patient’s perspective  *Sub-constructs:* Supportive behaviours, empathetic attitudes, verbal and non-verbal communication  *Underlying concepts:* Co-production, self-determination, personalised care, shared decision-making | Outpatient–community setting in Portugal | Focus group interviews | Content analysis | Patients emphasised that empathetic, respectful nurse–patient relationships and involvement in care decisions were vital to recovery. Personalised communication and recognition of individual needs fostered trust and were viewed as being pivotal for healing. | The study had a clear design, rigorous analysis, and strong ethics. It provided valuable insights into patient–nurse relationships, though a small, diagnosis-specific sample and single-site recruitment limited generalizability. |
| Donaghay-Spire et al. (2015), UK | To explore what happens when psychological input is offered in acute inpatient mental health settings and how service users and staff understand and portray those experiences | *Method:* Purposive sampling  *Channel:* Clinic patients and users; direct and indirect contact via networks, organisations and/or gatekeepers | 10 participants: 4 service users and 6 staff members— psychiatrists, nurses, social workers, and occupational therapists (OTs) | Examining the psychological aspects of acute inpatient care by focusing on recovery-oriented dialogue, therapeutic relationships, and psychological change and examining meaning-making, emotional processing, and inter- and intrapersonal aspects of the recovery process | Three inpatient hospitals and four community services in a single National Health Service (NHS) Mental Health Trust in the UK, plus acute inpatient mental health wards | Individual interviews | Narrative analysis, triangulation, member check | The study showed that psychological input in acute care supported recovery by fostering meaning, hope, and empowerment. Its impact, however, was shaped by service availability, patients’ level of engagement, contextual challenges, and access to care. | The study was methodologically intense and met all five qualitative criteria, which supported the credibility and relevance of its findings. Despite limited generalisability, it offered confident guidance for practice, policy, and future research on inpatient psychological care. |
| Eiroa-Orosa et al. (2025),  Spain | To analyse the group narratives and outcomes of the first trialogue meetings in Barcelona and explore their potential for transforming mental healthcare by promoting shared understanding and open dialogue among stakeholders | *Method:* Random sampling  *Channel:* Clinic patients and users; direct and indirect contact via networks, organisations, and/or gatekeepers | 13 participants: 9 people with lived experience of psychosocial distress and their relatives, 1 person identified solely as a relative, and 3 “MHPs (i.e. social worker, social educator, and psychologist)”  **Our synthesis excluded responses from relatives.* | The trialogue as a recovery-oriented, dialogical practice while emphasising equal participation, mutual learning, and open communication between people with lived experience and MHPs | Community mental health centre in Barcelona, Spain, involved in a transformational recovery-oriented care initiative | Treatment and intervention meetings | Thematic analysis | The study showed that trialogue meetings fostered recovery-focused dialogue through respectful, inclusive exchanges among users, families, and staff. Those initiatives supported CHIME principles, although challenges such as unclear roles and irregular attendance limited their consistency and impact. | The study demonstrated strong methodological and ethical integrity, with clear alignment across design and analysis. Despite minor issues with participants’ comprehension and demographic details, it offered valuable insights into trialogues as inclusive, recovery-oriented dialogue. |
| Faith et al. (2023), US | To examine how clinicians in two psychiatric treatment models— standard psychiatric rehabilitation and metacognitive reflection and insight therapy (MERIT)— conceptualise and speak about recovery and how it influences their practice and therapeutic conversations | *Method:* Purposive sampling  *Channel:* Direct and indirect contact via networks, organisations, and/or gatekeepers; personal and workplace referrals | 12 staff members: 6 who have worked in standard psychiatric rehabilitation settings and 6 trained MERIT therapists | Examining the language and meaning of recovery in therapeutic settings as well as collaborative dialogue, identity development, goal-directed care, and recovery orientation, with the central construct of recovery as conceptualised in clinical conversations | Outpatient psychiatric settings in the US, where some clinicians work in standard community mental health rehabilitation programmes, while others work in settings implementing MERIT psychotherapy, which emphasises personal meaning and metacognitive development | Direct and indirect contact via networks, organisations, and/or gatekeepers; personal and workplace referrals | Individual interviews | The study showed that MERIT clinicians used collaborative, meaning-focused dialogue to support identity and growth, while rehabilitation clinicians emphasised structured, goal-driven talk. Both aided recoveries, although MERIT more strongly reflected CHIME principles, including connection and meaning. | The study featured strong methodological alignment, rich interview data, and ethical rigour. Despite a small, model-specific sample, it offered valuable insights into how clinical models shape recovery-focused dialogue in psychiatric care. |
| Forchuk et al. (2021),  Canada | To evaluate clients’ perceptions of the benefits and potential adjustments to the implementation of the transitional discharge model (TDM), which supports individuals with psychiatric illness in transitioning from hospital to the community | *Method:* Purposive sampling  *Channel:* Clinic patients and users | 87 patients | Recovery-oriented peer support in the TDM and how structured peer relationships and post-discharge conversations facilitate personal recovery, connectedness, and emotional security | Canadian psychiatric inpatient hospitals and community peer support organisations; the TDM involved ongoing support and conversations between clients and peer supporters after discharge from the hospital | Focus groups, field notes | Thematic analysis, ethnographic approach, triangulation, member check | The study showed that the TDM supported recovery by facilitating easier hospital-to-community transitions through peer and staff support. It enhanced confidence and connection, though trust issues and communication breakdowns challenged implementation. | The study showed strong methodological and ethical integrity, with well-aligned design and analysis. Despite limited scope and generalisability, it offered credible insights into peer-supported, recovery-oriented dialogue during psychiatric discharge transitions. |
| Hammervold et al. (2022), Norway | To explore patients’ experiences with and considerations about participating in post-incident reviews (PIRs) after restraint events and how they perceive PIRs’ potential for care improvement and restraint prevention | *Method:*  Purposive sampling  *Channel:*  Direct and indirect contact via networks, organisations, and/or gatekeepers | 8 individuals who had experienced at least one restraint followed by a PIR during inpatient care | PIRs as a recovery-oriented intervention after coercive restraint events, explored via patients’ lived experiences | Two psychiatric settings in Norway that use coercive measures. | Individual interviews | Thematic analysis, phenomenological hermeneutical analysis, narrative analysis | The study showed that PIRs supported recovery by encouraging reflection, dialogue, and empowerment after coercive events. Their effectiveness relied on patient-centred approaches that addressed power dynamics and promoted CHIME principles through inclusive participation. | Meeting all MMAT 2018 qualitative criteria, the study had a strong design, ethical rigour, and rich analysis. Despite recruitment barriers from gatekeeping, it offered trustworthy insights into patients’ experiences with PIRs in psychiatric care. |
| Horgan et al. (2021),  Australia, New Zealand, Netherlands, Norway, Iceland, and Ireland | To explore mental health service users’ perspectives on the desired characteristics of mental health nurses to inform the development of a co-produced mental health nursing education module | *Method:* Purposive sampling  *Channel:* Flyers in clinics, community centres, and patient and user organisations | 50 individuals with lived experience of using mental health services and with diverse backgrounds from six countries | Examining desirable qualities of mental health nurses from the perspective of service users and linked to therapeutic relationships and recovery-oriented care | Community and academic settings across six countries, with focus groups conducted in universities or community centres | Focus groups | Thematic analysis, triangulation, member check | The study highlighted that empathetic, respectful, person-centred communication fostered therapeutic relationships and supported recovery. However, task-driven routines, stigma, and hierarchical dynamics in psychiatric care often undermined those interactions and limited their transformative potential. | Using co-produced focus groups and rigorous analysis, the study met all MMAT 2018 qualitative standards. Despite some recruitment constraints, it offered credible, cross-national insights into service users’ perspectives on recovery-oriented mental health nursing. |
| Hristodoulidis et al. (2022), Australia | To explore mental health nurses’ lived experience, understanding, and knowledge of personal recovery-oriented care (PRC) on acute mental health units | *Method:*  Purposive sampling  *Channel:* Flyers in clinics or community centres; personal and workplace referrals | 7 mental health nurses | PRC in acute psychiatric nursing while focusing on definitions, nurses’ understandings, and barriers to implementation | A locked acute mental health unit in a metropolitan hospital in Melbourne that included a low dependency area and an acute management area with nurses working across both areas and caring for involuntarily admitted patients | Individual interviews | Thematic analysis, triangulation, member check | The study showed that nurses often struggled to distinguish personal from clinical recovery, which limited recovery-focused dialogue. Supportive conditions enabled engagement, but time pressure, custodial roles, and medical dominance frequently hindered meaningful, person-centred conversations. | The study met all qualitative standards through a rigorous hermeneutic design and co-produced analysis. Although limited by a small, single-site sample, it provided rich, credible insights into nurses’ experiences with personal recovery-oriented care. |
| Hyde et al. (2015), Australia | To explore consumers’ lived experiences with inpatient mental health care and identify what they perceive as most helpful for recovery, with a focus on social work contributions within a recovery-oriented practice (ROP) framework | *Method:* Purposive sampling  *Channel:* Clinic patients and users | 8 patients | Examining consumers’ lived experience with acute inpatient care and its recovery-supporting elements (e.g. listening and validation, peer support, and family involvement) | A large rural acute mental health facility in New South Wales, Australia, operated under a dominant biomedical model in which consumers are often involuntarily admitted and subjected to medication and legal interventions | Individual interviews | Phenomenological hermeneutical analysis | The study showed that psychiatric inpatient care was often limited by clinical language and time, which left patients feeling unheard. Genuine listening, especially from peers or empathetic staff, fostered connection, meaning, and empowerment, thereby enhancing recovery and a sense of belonging. | The study demonstrated consumer coherence through hermeneutic phenomenology while exploring consumers’ lived experiences with inpatient care. Reflexivity and rich narratives strengthened it, though its small, rural Australian sample limit broader transferability. |
| Igarachi et al. (2024), Japan | To identify conversation topics in psychiatric outpatient consultations, determine who initiates them, and examine differences in topic content and initiation with and without a shared decision-making (SDM) tool | *Method:*  Purposive subset sampling  *Channel:* Clinic patients and users | 52 patients: 25 in the SDM group and 27 in the treatment-as-usual group | Recovery-oriented communication by exploring conversation topics and their initiation in psychiatric consultations | Outpatient psychiatric care in Japan, with and without the use of the SDM tool | Existing datasets, secondary data | Content analysis, Wilcoxon rank-sum tests | Patient-centred dialogues using tools such as  Shared decision–making helped patients to express goals and values, which fostered engagement, empowerment, and satisfaction. However, time constraints and demands of preparation challenged their implementation. | The study showed strong methodological rigour with clear questions, suitable qualitative methods, and coherent data alignment. Despite offering trustworthy insights, limitations included the absence of visual recordings, potential coding bias, and limited cultural transferability. |
| Isobel S (2019), Australia | To explore voluntary and involuntary service users’ experiences of inpatient mental health care and assess the feasibility of proactive, collaborative feedback mechanisms | *Method:* Convenience sampling  *Channel:* Clinic patients and users | 67 patients | Examining experiences in inpatient mental health care, recovery-oriented care, and service user involvement | Two acute adult mental health inpatient units within general hospitals in Sydney, Australia | Individual interviews, surveys, questionnaire | Thematic analysis, content analysis, descriptive analysis | The study showed that though some patients viewed staff as being approachable and hopeful, recovery-focused conversations were hindered by inconsistent communication, emotional insecurity, and fear. Many patients felt powerless, were excluded, and hesitated to speak openly. | The study demonstrated strong methodological quality across qualitative, quantitative, and mixed-methods approaches. It combined structured surveys with rich thematic analysis. While qualitative insights were robust, quantitative data were limited by the small sample size and a lack of inferential analysis. |
| Jørgensen et al. (2022),  Denmark | To develop a recovery-oriented model for network meetings that supports users’ personal recovery through improved cross-sectoral collaboration between mental health hospitals and community mental health services | *Method:*  Purposive sampling  *Channel:* Flyers in clinics or community centres; patient and user organisations; email invitations; phone invitations | 25 participants: 10 MHPs at hospitals (e.g. nurses, physicians, and OTs), 8 MHPs at community mental health services (e.g. pedagogues, nurses), and 7 users with lived experience of mental health services | Recovery-oriented conversations (ROCs) and network meetings, grounded in the CHIME framework and open dialogue, to support personal recovery | A cross-sectoral mental healthcare setting in Denmark involving collaboration between mental health hospitals and community mental health services, with a focus on network meetings following discharge | Workshops | Content analysis | The study demonstrated that user-centred, dialogical interactions based on the CHIME framework facilitated personal recovery beyond symptom management. Structured yet flexible meetings fostered collaboration, though time limits, medicalised practices, and fragmented care hindered implementation. | The study employed an action research design that incorporated participatory workshops and content analysis to co-develop a recovery model. While offering valuable insights, it lacked triangulation, data transparency, and broader transferability due to its Danish context. |
| Karbouniaris et al. (2022), Netherlands | To explore service users’ perceptions of MHPs’ use of experiential knowledge and how it contributes to personal recovery | *Method:*  Comprehensive sampling  *Channel:* Direct and indirect contact via networks, organisations, and/or gatekeepers | 22 service users receiving psychiatric care from MHPs trained in using experiential knowledge | Examining experiential knowledge of MHPs and their role in ROCs | Three mental health organisations in north-eastern Netherlands focused on recovery-oriented care, with professionals such as social workers, nurses, and counsellors trained in using their lived experience | Individual interviews, focus groups | Thematic analysis, triangulation, member check | The study showed how relational, compassionate dialogues, in which MHP shared their lived experiences, built trust and supported recovery. Those CHIME-based conversations were aided by training and openness but hindered by stigma, unclear boundaries, and rigid norms. | The study demonstrated strong methodological rigour through in-depth interviews, focus groups, and rich participant-driven analysis. Ethical and participatory approaches enhanced credibility, though generalizability and potential selection bias remained minor limitations. |
| Keefe et al. (2020), US | To explore barriers and facilitators experienced by individuals with serious mental illness (SMI) in establishing positive therapeutic relationships in long-term inpatient psychiatric settings | *Method:*  Purposive sampling  *Channel:* Clinic patients and users; direct and indirect contact via networks, organisations, and/or gatekeepers | 22 service users | Examining therapeutic relationships and both barriers and facilitators to establishing meaningful, recovery-oriented dialogue between providers and patients in psychiatric hospitals | Three state psychiatric hospitals providing long-term care for economically disadvantaged individuals with SMI (average stay = approx. 9.6 months) in the US | Individual interviews | Thematic analysis, triangulation, member check | The study revealed that therapeutic relationships built on empathy and collaboration supported recovery through CHIME principles. Those relationships were enabled by active listening and shared planning but challenged by time limits, poor communication, and systemic barriers. | The study presented a clear aim, suitable design, and rigorous thematic analysis of therapeutic relationships in state psychiatric hospitals. Rich findings and methodological coherence were strengths, whereas limitations were the sample’s scope and the absence of providers’ views. |
| Kehoe et al. (2023),  Australia | To explore consumers’ views and experiences of recovery-oriented practicse (ROPs) in community mental health services following staff training in the  REFOCUS– principles unite local services assisting  recovery (PULSAR) programme | *Method:*  Convenience sampling; purposive sampling  *Channel:* Clinic patients and users | 21 consumers | ROPs, specifically as perceived and experienced by consumers after staff training | Public community mental health services in Melbourne, Australia, where staff had received REFOCUS–PULSAR ROP training | Individual interviews | Thematic analysis, triangulation, member check | The study demonstrated that supportive, trust-building interactions fostered a sense of hope and meaning. While strong relationships and staff engagement facilitated recovery, many staff members did not recall discussing recovery concepts. Barriers included unclear language, reliance on medication, and limited time. | The study demonstrated methodological rigour through its participatory design, semi-structured interviews, and reflexive analysis. It offered rich, consumer-informed insights, though limited recall of recovery concepts highlighted a need for more transparent clinical dialogue. |
| Kidd et al. (2015),  Australia | To explore the meaning of recovery-oriented care and how consumers’ participation can support the development of recovery-oriented mental health services | *Method:*  Purposive sampling  *Channel:* Clinic patients and users; direct and indirect contact via networks, organisations, and/or gatekeepers; patient and user organisations | 11 participants: 6 consumers, 4 clinicians (i.e. nurses and doctors), and 1 carer  ** The carer’s perspective was excluded from our analysis*. | Recovery-oriented care, consumers’ participation, power dynamics, and co-production in mental health services | A regional psychiatric service in Australia in the early stages of implementing ROPs | Workshops, participatory action research | Thematic analysis | The study showed that collaborative dialogues centring lived experiences and challenging power dynamics promoted recovery. Storytelling, co-led workshops, and strengths-based communication helped foster CHIME principles, although systemic barriers limited broader service transformation. | The study employed rigorous methodology, involved an in-depth thematic analysis, and facilitated meaningful stakeholder engagement. Despite a small, localised sample that limits its generalisability, its findings offered valuable insights for transforming mental health care services. |
| Kirkegaard Thomsen et al. (2024), Denmark | To examine how MHPs conceptualise personal recovery, narrate their professional contributions to recovery, and describe the personal and professional benefits of supporting recovery-oriented care | *Method:*  Purposive sampling  *Channel:* Email invitations; personal and workplace referrals | 48 staff members: 20 nurses, 10 psychologists, 4 psychiatrists, 7 social and health care assistants, 2 peer support workers, 2 activity workers, 1 social worker, and 2 physiotherapists | Personal recovery and recovery-oriented care, with an emphasis on how staff understand and contribute to it and recovery work’s impact on MHPs | A psychiatric hospital in Denmark with inpatient and outpatient services | Written feedback, surveys, questionnaires | Interpretative phenomenological analysis | The study revealed that conversations in trusting relationships helped patients to process emotions, build hope, and pursue goals and fostered recovery and staff motivation, though time pressures, workloads, and clinical priorities hindered them. | The study was methodologically sound, with well-supported themes and validated coding. However, reliance on written narratives, a sample of predominantly women nurses, and a single-site context limited depth and transferability. Even so, it provided credible insights into recovery care. |
| Klevan et al. (2024),  Norway | To explore the characteristics of experiences in mental health recovery-oriented places and how the characteristics can facilitate social connections and participation | *Method:*  Purposive sampling  *Channel:* Flyers in clinics or community centres; direct and indirect contact through networks, organisations, and/or gatekeepers; personal and workplace referrals | 16 participants in 8 dyads, each including a mental health service user and an MHP from mental health and substance use services | Recovery-nurturing places and their role in mental health recovery through material, social, and affective resources | A Norwegian municipality with recovery-oriented services, day centres, homes, and community locations (e.g. grocery stores and parks) | Individual interviews; participant observation | Content analysis | The study showed that psychiatric care often emerged informally through everyday dialogues in safe, shared spaces, sparked by familiar activities. Those interactions supported recovery by fostering CHIME principles through inclusive and meaningful engagement. | The study was methodologically sound due to integrating theory, practice, and lived experience to guide service development. Despite the limited sampling details and context specificity, its depth and relevance made it a strong qualitative contribution to mental health recovery. |
| Lauzier-Jobin and Houle (2024), Canada | To explore and describe the mechanisms of the helping relationship that promote personal recovery by analysing the perspectives of both persons in recovery and MHPs  using a critical realist approach | *Method:*  Snowball sampling; purposive sampling  *Channel:* Social media posts; flyers in clinics or community centres; patient and user organisations; phone invitations | 30 participants: 15 individuals in recovery and 15 MHPs (i.e. psychologists, social workers, nurses, OTs, and community workers) | Key mechanisms of the helping relationship that promote personal recovery, including communication, emotional support, presence, trust, problem-focused support, influence, and transcending the professional role | Mental health service context in community, public, and private outpatient settings in Canada focusing on recovery support outside institutional inpatient care, including therapeutic and psychosocial interventions | Individual interviews, surveys, questionnaire | Thematic analysis, triangulation, member check | The study showed that trust-based, collaborative interactions facilitated recovery through the presence, empathy, and flexibility that transcended traditional roles. Institutional norms, power imbalances, and unclear guidance on reciprocity hindered those CHIME-driven dialogues. | The study showed strong methodological quality using a critical realist framework, triangulated perspectives, and thematic analysis. Despite limited demographic diversity and generalizability, it offered credible insights into person-centred recovery-oriented care. |
| Molin et al. (2020),  Sweden | To illuminate staff’s experiences of introducing and participating in the time together (TT) nursing intervention in psychiatric inpatient care | *Method:*  Purposive sampling  *Channel:* Direct and indirect contact via networks, organisations, and/or gatekeepers; personal and workplace referrals | 17 staff members: 8 enrolled nurses, 8 registered nurses (RNs), and 1 OT | TT as a concept for use in mental health nursing settings and focused on staff–patient interaction, reciprocal conversation, engagement, and supporting recovery | Three locked psychiatric inpatient units in northern Sweden: two for substance-related conditions and one for acute mental health, serving voluntary and involuntary adults, with stays lasting 5–14 days and a high workload, medical focus, and limited nursing structure pre-TT | Individual interviews | Content analysis | The study showed that TT fostered ROCs through shared activities and relaxed staff–patient interactions. Those CHIME-based dialogues built trust and connection but faced challenges, including time limits, differences in care philosophy, and staff resistance. | The study demonstrated high methodological quality due to using semi-structured interviews and content analysis to explore staff experiences with TT. Despite its regional focus, it provided rich, credible insights into recovery-oriented psychiatric nursing. |
| Pelto-Piri et al. (2019), Sweden | To enhance understandings of how psychiatric inpatients perceive feelings of safety and unsafety in the ward environment | *Method:*  Purposive sampling  *Channel:* Clinic patients and users; direct and indirect contact via networks, organisations, and/or gatekeepers | 17 patients | Patients’ perceptions of safety and unsafety in psychiatric inpatient care, communication with staff, ward routines, social climate, and patient–staff dynamics *Constructs:* Emotional safety, powerlessness, therapeutic communication, organisational structure | Four psychiatric inpatient clinics in Sweden, including general, addiction, and forensic psychiatry units with varying levels of security | Individual interviews | Thematic analysis, content analysis | A ROCA fostered emotional safety, trust, and empowerment when staff were communicative and present. Supportive dialogue and shared responsibility enhanced recovery, while disengagement, delays, and limited interaction posed barriers. | The study demonstrated strong methodological rigour with a clear aim, appropriate semi-structured interviews, and well-executed thematic analysis. Supported by participants’ quotations, it offered credible insights into patient safety, despite its specific context. |
| Pfeiffer et al. (2019),  Sweden | To explore and highlight how patients with a dual diagnosis experience conversations with nurses in an outpatient clinic as being caring | *Method:* Purposive sampling  *Channel:* Clinic patients and users; direct and indirect contact via networks, organisations, and/or gatekeepers | 5 outpatient patients | Caring conversations while incorporating relational, narrative, and ethical communication | Outpatient addiction clinic in Sweden within a psychiatric care framework | Individual interviews | Phenomenological hermeneutical analysis | The study demonstrated that caring interactions rooted in relational, narrative, and ethical communication fostered safety, dignity, and self-esteem. Those conversations supported personal recovery through CHIME principles but were hindered by stigma, time limits, and organisational barriers. | The study demonstrated methodological rigour, strong theoretical grounding, and rich data interpretation. Although its small, purposively selected sample limit generalisability, it provided valuable insights into the relational and ethical aspects of recovery-oriented psychiatric conversations. |
| Prytz et al. (2019),  Norway | To explore how therapists experience negotiating a working alliance with patients with SMIs subjected to involuntary treatment | *Method:*  Snowball sampling  *Channel:* Clinic users and patients; personal and workplace referrals | 10 experienced clinicians: 4 clinical psychologists and 6 psychiatrists | Working alliances in involuntary psychiatric treatment through the lens of ROP and the tension between autonomy and coercion | Public psychiatric hospitals in western Norway, specifically in acute inpatient, long-term inpatient, and outpatient settings, that involve the involuntary treatment of patients with SMIs | Individual interviews | Thematic analysis, interpretative phenomenological analysis | The study revealed that conversations in psychiatric care often struck a balance between patient autonomy and clinical enforcement. While trust and inclusion were encouraged, shared decision-making was limited, and recovery principles, including empowerment and choice, were only partly achieved. | The study demonstrated strong coherence across research questions, methodology, and analysis, with well-supported findings. However, its reliance on therapists’ self-reports and lack of patients’ perspectives limited the depth of insight into therapeutic alliances. |
| Raitakari et al. (2018), Finland | To identify and categorise the elements of the “recovery in” model (RIM) and analyse how the elements are constructed in positive assessment sequences during mental health home visit interactions | *Method:*  Purposive sampling  *Channel:*  Clinic patients and users; direct and indirect contact via networks, organisations, and/or gatekeepers; personal and workplace referrals | 10 patients | RIM assessments in client–MHPs conversations as expressions of recovery-oriented support | Mental health floating support services provided in clients’ homes, operated by a local non-profit with a recovery-oriented philosophy, and reflecting a community-based, deinstitutionalised approach to psychiatric care | Clinical interactions, real-life conversations | Ethno-methodological interaction research | The study was grounded in the RIM, where MHPs used positive assessments during home visits to support clients’ agency, growth, and community integration. Those conversations fostered hope and empowerment but were shaped by cultural norms and risked reinforcing conformity if not carefully personalised. | The study demonstrated rigour in its approach, data collection, coding, and analysis. It effectively explored how MHPs-client dialogues enacted recovery-oriented elements. Despite a small sample and lack of client-reported experiences, the authors acknowledged those limitations. |
| Reed et al. (2018),  Norway | To explore how community mental health workers provide support to users by investigating MHPs narratives of how they work in recovery-oriented ways | *Method:*  Purposive sampling  *Channel:* Personal and workplace referrals | 7 staff members: 3 nurses, 1 social worker, 1 OT, 1 sociologist, and 1 practical nurse | Negotiating support in recovery-oriented community mental health work | Urban community-based mental health services in Norway focused on everyday life support and home-based care | Individual interviews | Interpretive analysis | The study identified conversations as flexible, negotiated interactions balancing user autonomy with professional duties in daily life. Those supported CHIME principles —trust, identity, meaning, and empowerment —but faced challenges from systemic constraints and power imbalances. | The study demonstrated strong alignment between its research question, narrative methodology, and interpretive analysis. Rich interview data and thoughtful thematic interpretation supported its findings. Despite a small, MHPs-only sample, it offered valuable insights into recovery-oriented community mental health practice. |
| Reinius et al. (2023),  Sweden | To explore how patients in inpatient mental healthcare experience the intervention of daily talks, a patient-driven innovation aimed at enhancing meaningful interactions between patients and staff | *Method:*  Purposive sampling  *Channel:* Clinic patients and users; direct and indirect contact via networks, organisations, and/or gatekeepers | 14 patients | Daily talks (i.e. a structured, patient-led conversation with staff) aimed at enhancing recovery through meaningful interaction | Psychiatric inpatient acute care ward in a hospital in Sweden | Individual interviews | Thematic analysis | Daily talks empowered psychiatric inpatients through patient-led conversations with staff that enhanced trust, connectedness, and recovery. Those simple, routine-friendly interactions supported autonomy, emotional expression, and collaborative, person-centred care. | The study had a clear aim, employed a qualitative design, and used reflexive thematic analysis, which enhanced its credibility. Ethical practices and purposive sampling supported the findings, although limited demographics and single-site recruitment reduced generalisability across broader patient populations. |
| Rooney et al. (2016), UK | To explore inpatients’ experiences with intentional mental health peer support on psychiatric wards | *Method:* Volunteer sampling; purposive sampling  *Channel:* Flyers in clinics or community centres | 7 patients | Intentional peer support in mental health settings  *Key phenomenon:* Patients’ lived experiences and perceptions of peer support during inpatient psychiatric care | Two inpatient psychiatric wards (i.e. one acute, one recovery-focused) within Worcestershire Health and Care of the NHS Trust, UK | Individual interviews | Thematic analysis | The study showed that psychiatric care was supported by intentional peer support, which fostered person-centredness, hope, and emotional and practical help. Those interactions reflected CHIME principles by promoting identity, meaning, empowerment, and connection through shared, non-judgemental experiences. | Though the study had a research aim, it did not seem to reflect a qualitative design and could easily be mistaken for quantitative research. In that regard, the analytical method and results lacked sufficient detail, which affected the qualitative focus applied. However, the results align with our research aim, hence its inclusion. |
| Sellin et al. (2018), Sweden | To describe what characterises a recovery-oriented caring approach (ROCA) and how it can be expressed through caring acts involving suicidal patients and their relatives | *Method:*  Purposive sampling  *Channel:* Direct and indirect contact via networks, organisations, and/or gatekeepers; patient and user organisations; personal and workplace referrals | 16 participants in three expert groups: 5 representatives from a Swedish suicide prevention organisation (i.e. experts by lived experience), 6 RNs, and 5 researchers with expertise in suicide prevention | Recovery-oriented care focused on communicative togetherness, a relational, dialogical process that supports suicidal patients through sensitive listening, shared reflection, and the acknowledgement of their lived experiences | Swedish mental health care context, with a focus on psychiatric inpatient care for individuals with suicidal ideation | Focus groups, Delphi method, surveys, questionnaires | Thematic analysis | The study identified “communicative togetherness” as a relational process that supported suicidal patients through sensitive listening and shared reflection. It fostered hope, identity, and empowerment by enabling expression, reconnection, and active participation in recovery. | The study demonstrated strong methodological rigour through a clear aim, appropriate use of the Delphi method, and expert-informed thematic analysis. Despite a small, homogeneous sample and a lack of patients’ voices, it offered relevant insights for recovery-oriented psychiatric care. |
| Selvin et al. (2021), Sweden | To describe MHPs’ perceptions of the concept of patient participation in forensic psychiatric care | *Method:*  Strategic sampling  *Channel:* Direct and indirect contact via networks, organisations, and/or gatekeepers; personal and workplace referrals | 19 staff members: 5 nursing assistants, 7 nurses, 2 counsellors, 2 psychologists, and 3 psychiatrists | Patients’ participation in forensic psychiatric care | Two medium-security forensic psychiatric clinics in Sweden | Individual interviews | Phenomeno-graphic analysis | The study identified that collaborative, individualised dialogues fostered patient involvement, understanding, and empowerment. Supported by staff teamwork and tailored care, those conversations promoted recovery principles but were hindered by bureaucracy, communication gaps, and patient-specific challenges. | The study demonstrated methodological rigour through a suitable phenomenographic approach, coherent design, and findings grounded in MHPs views. Despite the risk of researcher bias, lack of patient input, and limited generalisability, it offered credible insights into coercive care. |
| Shue et al. (2023), US | To examine factors affecting the implementation of recovery-oriented inpatient treatment planning in mental health units | *Method:*  Purposive sampling  *Channel:* Clinic patients and users; direct and indirect contact via networks, organisations, and/or gatekeepers | 53 staff members (i.e. nurses, psychologists, social workers, psychiatrists, local recovery coordinators, and other staff) across 13 inpatient mental health units, 3 of whom were key informants | Implementation of recovery-oriented inpatient treatment planning | Acute inpatient mental health units in the US under the Veterans Health Administration | Individual interviews, participant observation, field notes | Thematic analysis, grounded theory | The study showed that collaborative, person-centred dialogues focused on long-term goals and that shared decision-making supported patient inclusion in care. Successful implementation relied on staff training and culture, though barriers included access to therapy and the dominance of prescribers. | The study demonstrated methodological rigour through clear research alignment, use of normalisation process theory, and triangulated data. Though limited to Veterans Health inpatient units, it offered credible insights for broader recovery-oriented care. |
| Solomon et al. (2021), New Zealand | To explore the experience and meaning of ROP for nurses working in an acute inpatient mental health service | *Method:*  Volunteer sampling  *Channel:* Flyers in clinics or community centres; patient and user organisations; open invitation | 10 RNs | Recovery-oriented nursing practice, especially how nurses understand and enact it by creating safe, shared, and healing relational spaces | An acute inpatient mental health service in New Zealand | Individual interviews | Thematic analysis, phenomenological hermeneutical analysis | The study showed that nurses created safe, healing environments by fostering trust, validating patients’ experiences, and promoting autonomy and hope. Those practices, though challenged by systemic barriers, were strengthened by reflection, cultural responsiveness, and supportive environments. | The study employed a strong phenomenological approach, rigorous thematic analysis, and credible methods, including reflexive diaries and peer review. Despite a small, single-site sample and a lack of service user input, it offered robust insights into recovery-focused nursing. |
| Twamley et al. (2021), Ireland | To explore the lived experiences of service users and their support networks participating in an open dialogue (OD)-informed mental health service by examining how the approach impacts relationships, communication, and perceptions of care | *Method:*  Convenience sampling  *Channel:* Clinic patients and users; direct and indirect contact via networks, organisations, and/or gatekeepers | 22 individuals from 10 networks: 12 service users and 10 partners, parents, and friends  **Data from the network members were excluded from our synthesis.* | OD-informed ROCs and how dialogic practices (e.g. shared reflections, inclusion, and polyphony) shape service users’ experiences | A rural community mental health team in Ireland implementing OD alongside treatment-as-usual (i.e. psychiatric assessment and professional multidisciplinary team decisions) | Individual interviews | Thematic analysis | The study showed that OD-based conversations prioritised trust, shared understanding, and flexible, transparent practices, which fostered recovery, though some participants struggled with self-reflection and uncertainty or preferred structured problem-solving approaches. | The study was methodologically robust, with validated themes and triangulated data. Despite a small, self-selected sample and the early-stage use of OD, it offered credible insights into how dialogic conversations enhance relationships and recovery. |
| Vandewalle et al. (2019), Belgium | To uncover and understand the core elements of how nurses in psychiatric hospitals make contact with patients experiencing suicidal ideation | *Method:*  Purposive sampling  *Channel:* Direct and indirect contact via networks, organisations, and/or gatekeepers; email invitations | 19 psychiatric nurses | Nurse–patient contact in the context of suicidal ideation  *Key phenomena:* Conditions for open, genuine communication and developing an accurate, meaningful picture of patients | Four psychiatric hospitals in Flanders, Belgium, specifically adult psychiatric wards that regularly admit patients with suicidal ideation | Individual interviews | Grounded theory, qualitative analysis guide of Leuven (i.e. QUAGOL) | The study showed that compassionate, trust-building conversations prioritised understanding the person behind suicidal thoughts. Supported by emotional engagement and balanced procedures, the conversations fostered recovery, whereas clinical demands and systemic constraints challenged them. | The study demonstrated high methodological quality by meeting MMAT criteria through the use of grounded theory and a coherent design. Despite lacking patients’ perspectives and limited generalisability, it offered robust insights within Belgium’s specific cultural and institutional context. |
| van Lankeren et al. (2020), Netherlands | To gain insight into the experiences of the nursing care provided for patients diagnosed with a bipolar Ι disorder and hospitalised in a closed ward for an acute manic episode | *Method:*  Purposive sampling  *Channel:* Clinic patients and users; direct and indirect contact through networks, organisations, and/or gatekeepers | 12 patients | Patients’ experiences with nursing care during involuntary psychiatric hospitalisation for mania | Closed psychiatric wards in three mental health organisations in the Netherlands. | Individual interviews, field notes | Thematic analysis, Stevick–Colaizzi–Keen method, triangulation, member check | The study highlighted the importance of respectful, calm communication in fostering safety and emotional regulation during mania. Facilitators included nurse visibility and routines, whereas unclear goals, rigid rules, and novice staff posed barriers; however, there were opportunities for reflection after distressing events, including seclusion. | The study was well-designed and executed, with appropriate methods and thoughtful analysis. While limitations such as potential selection bias and low member check engagement were acknowledged, they did not significantly undermine the credibility of the findings. |
| Waldemar et al. (2019), Denmark | To explore how ROP is reflected in interactions between patients and MHPs around treatment in mental health inpatient wards | *Method:*  Purposive sampling  *Channel:* Flyers in clinics or community centres; direct and indirect contact via networks, organisations, and/or gatekeepers | >45 participants (some participants were not counted): patients, 36 RNs and nurse assistants, 2 psychiatrists, 2 psychologists, 3 secretaries, 1 recovery mentor, and additional medical doctors, students, social workers, and physiotherapists | Examining ROPs in inpatient psychiatric care  *Key phenomenon:* Patients–MHP interactions, particularly in treatment planning and decision-making  *Observation focus:* Interactions during ward rounds, consultations, group sessions, informal conversations, and daily routines | Two adult psychiatric inpatient wards (open and locked) in a public hospital in Denmark offering early-stage implementation of ROPs, including staff training and environmental changes | Individual interviews, field notes | Content analysis, ethnography | The study showed that inpatient settings often invited patient input without influencing decisions, which remained staff-led. Principles of recovery were acknowledged but frequently overridden by routines, power imbalances, and competing institutional demands. | The study employed rigorous ethnographic methods with rich data and coherent analysis. However, limited private observations, minimal peer support engagement, and a focus on two wards resulted in limited insight into interpersonal dynamics and broader applicability. |
| Walde et al. (2023), Germany | To explore forensic mental health patients’ perspectives on the implementation of a peer support worker in a forensic psychiatric hospital by focusing on their experiences, acceptance, and perceived effects of the intervention | *Method:*  Purposive sampling  *Channel:* Clinic patients and users; direct and indirect contact via networks, organisations, and/or gatekeepers | 18 patients | Peer support work in forensic psychiatry as a recovery-oriented intervention | Forensic psychiatric hospital at Rostock University Medical Centre in Germany, which treats offenders with substance use disorders | Individual interviews, focus groups | Individual interviews, focus groups | The study showed that empathetic, trust-based communication, shaped by shared lived experiences, enabled peer support workers to foster hope and empowerment, which helped patients to feel understood and engaged in their recovery within a supportive, non-hierarchical setting. | The study employed rigorous qualitative methods to investigate patients’ experiences with peer support in forensic psychiatry; however, self-selection bias, limited minority representation, and a specific setting reduced the generalisability of its findings. |
| Wallace et al. (2016), UK | To explore service users’ experiences of receiving the REFOCUS intervention, a complex, pro-recovery intervention designed to promote recovery-supporting tools and relationships on community mental health teams | *Method:*  Random sampling; convenience sampling; purposive sampling  *Channel:* Clinic patients and users; direct and indirect contact via networks, organisations, and/or gatekeepers | 37 service users | Service users’ experience in a recovery-oriented intervention (i.e. REFOCUS) | Community mental health teams in two NHS Trusts (i.e. South London and Maudsley and the 2gether NHS Foundation Trust) in the UK | Individual interviews, focus groups | Thematic analysis | The REFOCUS intervention fostered collaboration, self-awareness, hope, and a sense of identity. However, its impact varied; the rigid or insincere use of tools led some participants to find the communication to be impersonal and less effective. | The study used rigorous methods to explore service users’ experiences in a recovery-focused intervention. Credibility was supported by thematic analysis and participants’ quotations; however, biases and limited generalizability may affect the interpretation of findings. |
| Whittle et al. (2024), UK | To investigate how recovery orientation is nurtured and maintained in inpatient psychiatric rehabilitation and what factors facilitate or constrain it | *Method:* Purposive sampling  *Channel:* Clinic patients and users; direct and indirect contact via networks, organisations, and/or gatekeepers | 23 participants: 9 patients and 14 staff members (i.e. healthcare assistants, nurses, doctors, therapists, and managers) | Relational engagement as a core mechanism of recovery-oriented care | Apollo Ward, a 16-bed NHS psychiatric rehabilitation ward in London that serves patients with complex psychoses | Individual interviews, participant observation | Grounded theory, ethnography | The study revealed relational interactions built on respect, emotional connection, and recognition of progress. However, institutional demands, including risk management and staff burnout, often undermined their effectiveness by prioritising administrative over human care aspects. | The study showed high methodological quality, characterised by clear questions, an ethnographic design, and a coherent analysis. Relational insights were strong, though a single-site, narrow sample, and researcher bias limited broader applicability. |
| Zetterström et al. (2023), Sweden | To describe nurses’ experiences with using anxiety communication notes in the nursing relationship with patients suffering from anxiety in mental health inpatient care | *Method:*  Purposive sampling  *Channel:* Direct and indirect contact via networks, organisations, and/or gatekeepers; email invitations | 12 staff members: mental health nurses, general nurses, nursing assistants, and one with a background in human sciences | Anxiety communication notes as a structured, collaborative tool to support anxiety management, enhance nurse–patient communication, and empower patients | Two general mental health inpatient care wards in public hospitals in southwestern Sweden that care for adults with various psychiatric diagnoses | Individual interviews | Content analysis | The study showed that structured dialogues using anxiety notes fostered understanding, empowerment, and stronger bonds between nurses and patients. However, institutional routines, nurse resistance, and communication problems often hindered their effectiveness. | The study showed strong methodological quality characterised by a coherent design and justified methods. Relational insights were robust, though the lack of pilot testing and participants’ familiarity introduced bias, which was mitigated by careful analysis and researcher neutrality. |
| Quantitative Studies | | | | | | | | | |
| Howell et al. (2023), US | To examine whether aspects of social functioning (i.e. social engagement, interpersonal communication, and satisfaction with support) mediate the relationship between psychiatric symptoms and personal recovery among people with SMIs | *Method:* Purposive sampling  *Channel:* Clinic patients and users | 250 patients | Personal recovery in SMI and the mediating role of social functioning (i.e. support satisfaction, communication, and engagement) | Outpatient psychiatric services in the Veterans Affairs healthcare system and a community mental health centre as a clinical care context for adults with long-term SMIs | Surveys, questionnaire, clinical assessment, clinical history | Mediation analysis, regression analysis | The study showed that social functioning, especially communication and support satisfaction, mediated the link between psychiatric symptoms and recovery. Findings supported the integration of social skills training and holistic assessment to foster empowerment and connectedness despite ongoing symptoms. | The study employed a sound quantitative design by using validated tools and mediation analysis. However, generalisability was limited by a predominantly male veteran sample and unreported non-response rates, despite meeting most MMAT criteria. |
| Okumura and Katsuki (2024), Japan | To examine factors of effective communication for personal agency in recovery by investigating the association between perceived support through communication and the agency of individuals with mental illness | *Method:*  Convenience sampling  *Channel:* Clinic patients and users | 222 users of mental health services | Personal agency in mental health recovery, which involves effective provider communication as a facilitator of ROPs | Community-based psychiatric care in Japan, with a cultural emphasis on familiar, harmonious relationships, and therapeutic partnerships | Validated scales, surveys, questionnaires | Descriptive statistics, *t* tests, Analysis of Variance (ANOVA), Pearson and Spearman correlation, regression analysis | The study showed that emotionally attuned, partnership-based dialogues supported agency, decision-making, and life planning, which aligned with CHIME principles. Facilitators included therapeutic relationships and cultural sensitivity, while stigma and lack of frameworks posed barriers. | The study met key criteria for quantitative descriptive research, with clear aims, valid tools, and sound analysis. However, convenience sampling and a limited scope reduced its representativeness, introduced some nonresponse bias, and limited generalisability. |
| Pfeiffer et al. (2019), US | To develop and pilot test the feasibility, acceptability, and fidelity of the Peers for Valued Living (PREVAIL) peer support intervention for suicide prevention among psychiatric inpatients at high risk for suicide | *Method:*  Random sampling; purposive sampling  *Channel:* Clinic patients and users; direct and indirect contact via networks, organisations, and/or gatekeepers | 70 psychiatric inpatients: 34 in the intervention group and 36 in the usual care group | Suicide prevention through peer support  *Key phenomena:* Hope, connectedness, safety, recovery-oriented peer relationships *Constructs:* Hopelessness, belongingness, suicidal ideation, peer support fidelity | Inpatient psychiatric units and community-based follow-up over 12 weeks post-discharge. *Intervention:* PREVAIL, a peer support program, that includes structured tools and flexible delivery formats (i.e. in-person, phone, and text) in the US | Individual interviews, validated scales, clinical assessment, clinical history | Thematic analysis, descriptive statistics | The study showed that peer-led, flexible dialogues enhanced hope, safety, and connectedness in suicidal patients. Grounded in lived experience and motivational interviewing, they were feasible and valued for balancing empathy, advice, and shared recovery stories. | The study demonstrated a strong randomised controlled trial design, participant engagement, and qualitative rigour. Although the small sample size, lack of blinding, and single-site recruitment limit its generalisability, it laid valuable groundwork for future research. |
| Wong et al. (2019), US | To examine whether two aspects of provider communication (i.e. showing respect and explaining understandably) are associated with personal recovery outcomes (i.e. connectedness, hope, empowerment, life satisfaction, and internalised stigma) and whether those associations differ by provider type (i.e. MHP vs. general medical doctor) | *Method:* Stratified sampling; purposive subset sampling  *Channel:* Random digit dialling; registry | 429 patients | Respect and clarity in providers’ communication and CHIME-aligned personal recovery outcomes, along with life satisfaction and internalised stigma | Community-based, non-clinical population sample in California, US | Surveys, questionnaires, existing datasets, secondary data | Regression analysis | The study showed that respectful provider communication was strongly linked to improved recovery outcomes such as hope, empowerment, and reduced stigma, especially from MHPs. Those dialogues reflected CHIME principles and supported patient-centred psychiatric care. | The study demonstrated high methodological quality with clear aims, representative sampling, validated measures, and sound analysis. Despite a moderate response rate (45.2%), which introduced some bias, it provided strong evidence linking respectful communication to outcomes of recovery. |
| Mixed-Method Studies | | | | | | | | | |
| Banfield and Forbes (2018), Australia | To evaluate the processes and outcomes of the Partners in Recovery initiative in the Australian Capital Territory, a programme established to improve the coordination of health and social care for the studied population | *Method:* Comprehensive sampling with purposive subset sampling  *Channel:* Clinic patients and users; internal surveys; direct and indirect contact via networks, organisations, and/or gatekeepers | 39 participants who completed questionnaires (i.e. 25 clients and 14 service providers) and 6 clients and 4 service providers who participated in the interviews | Person-centred care coordination for complex mental health needs while highlighting the support facilitators’ role in ensuring continuity | Australian mental health care system, in the context of the Partners in Recovery programme, which includes community-based mental health services and health and social care services connected to the programme in the Canberra region | Individual interviews, written feedback, surveys,  questionnaires | Descriptive statistics, content analysis | The study showed that collaborative, person-centred dialogues, grounded in trust, shared decision-making, and holistic support, were crucial. Support facilitators played a key role by building relationships, empowering clients, and tailoring care to individual needs. Barriers included poor interagency communication and information sharing, while facilitators included strong rapport, proactive coordination, and a modern understanding of mental illness. | The article presented a robust mixed-methods evaluation with strong qualitative work and a moderately solid survey. Despite limitations in survey validation and response bias, it offered valuable insights into mental health care coordination and practice. |
| Molin et al. (2018), Sweden | To evaluate the feasibility and effects of the TT nursing intervention in psychiatric inpatient care | *Method:*  Convenience sampling  *Channel:* Clinic patients and users; open invitation | 125 participants: 80 patients and 45 staff members (i.e. RNs, enrolled nurses, OTs and unit managers) with varying levels of experience and training | TT, a structured nursing intervention based on ordinary joint activities designed to improve interaction quality, reduce stress, and support personal recovery | Three locked psychiatric inpatient units in two Swedish county councils that specialise in addiction or acute psychiatric care, with highly medical routines and limited psychosocial interventions | Participant observation, validated scales | Content analysis, visual trend analysis | The study demonstrated strong qualitative and mixed-methods rigour, with clear questions and integrated data. Despite the use of a non-validated scale and a modest response rate, the study effectively explored recovery communication in forensic mental health care. | The study offered strong qualitative insights into relational dynamics and contextual enablers. Despite missing data and inconsistent quantitative implementation, the integration was well-justified, and the interpretation was practical, which enhanced the study’s overall contribution. |
| Scheirich et al. (2024), Australia | To test a recovery-oriented communication script for forensic mental health nurses and explore whether including empathic statements affects nurses’ perceptions of empathy and the script’s potential to prevent aggression | *Method:*  Purposive sampling  *Channel:* Direct and indirect contact via networks, organisations, and/or gatekeepers; email invitations; personal and workplace referrals | 54 nurses | Recovery-oriented communication in forensic mental health nursing by focusing on nurses’ perceptions of empathy and aggression prevention in scripted dialogues, with measured constructs including hope, collaboration, choice, identity, safety, and procedural justice | Thomas Embling Hospital, a secure forensic mental health facility in Victoria, Australia | Validated scales, written feedback, surveys, questionnaires | Content analysis, Wilcoxon rank-sum tests | The study showed that structured, principle-based dialogues promoted empathy, collaboration, and patient involvement in aggression prevention. Nurses found them to be effective, though mental state and scripted communication complexity posed notable challenges. | The study demonstrated strong qualitative and mixed-methods rigour by integrating data effectively. While the quantitative part was limited by a non-validated scale and modest response rate, it nevertheless supported a meaningful exploration of recovery-oriented communication. |
| Sellin et al. (2019), Sweden | To explore and evaluate how a ROCA was experienced by a suicidal patient in the context of their close relatives and nurses | *Method:*  Purposive sampling  *Channel:* Clinic patients and users; direct and indirect contact via networks, organisations, and/or gatekeepers | 5 participants: 1 patient, 1 relative, and 3 nurses  **Data and findings from relatives were excluded from our synthesis.* | The ROCA model, which promotes patient-centred dialogue and mutual understanding as ways to support suicidal patients’ recovery | Psychiatric inpatient care in Sweden | Individual interview, validated scales, diaries or journals, clinical assessment, clinical history | Hermeneutic analysis | The study showed that the ROCA model fostered person-centred dialogue that supported connectedness, hope, and empowerment. Structured tools and trust enabled those benefits, though institutional routines, emotional strain, and time constraints often hindered meaningful interaction. | The study offered substantial qualitative depth and integration in providing a human-centred evaluation of the ROCA model. However, its single-case design and lack of statistical analysis limited the quantitative findings, as the authors acknowledged. |
| *Note.* ANOVA= Analysis of Variance; BPD = borderline personality disorder; CHIME = connectedness, hope, identity, meaning, and empowerment (i.e. principles used in recovery-oriented frameworks); DBT = dialectical behaviour therapy; MERIT = metacognitive reflection and insight therapy; MHP = mental health professional; MMAT = mixed methods appraisal tool; NHS = National Health Service; OD = open dialogue; OT = occupational therapist; PCC = population, concept, context; PIR = post-incident review; PULSAR= principles unite local services assisting recovery; PREVAIL= peers for valued living; RIM = “recovery in” model; RN = registered nurse; ROC = recovery-oriented conversation; ROCA = recovery-oriented caring approach; ROP = recovery-oriented practice; SDM= shared decision making; SMI = serious mental illness; TDM = transitional discharge model; TT = time together. | | | | | | | | | |
