# Supplementary material for: Recovery‐Oriented Conversations in Psychiatric Care: An Integrated Systematic Review
Source: Int J Ment Health Nurs. 2026 Jun 22;35(3):e70295. doi: 10.1111/inm.70295 (PMC13284807; doi:10.1111/inm.70295)
Supplement: Supplementary file 2 — Table S2: Quality appraisal of included articles using the Mixed Methods Appraisal Tool (MMAT). [file INM-35-0-s002.docx]

### **Supplementary Table 1.2**

Quality appraisal of included articles using the Mixed Methods Appraisal Tool (MMAT).

|  | **Study categories and MMAT criteria** | | | | | | | | | | | | | | | | | | | | | | | | | | |
| --- | --- | --- | --- | --- | --- | --- | --- | --- | --- | --- | --- | --- | --- | --- | --- | --- | --- | --- | --- | --- | --- | --- | --- | --- | --- | --- | --- |
|  | **Screening questions** | | **Qualitative** | | | | | **Quantitative randomised controlled trials** | | | | | **Quantitative non-randomised** | | | | | **Quantitative descriptive** | | | | | **Mixed methods** | | | | |
| **Article** | **S.1^‡^** | **S.2^‡^** | **1.1^§^** | **1.2^§^** | **1.3^§^** | **1.4^§^** | **1.5^§^** | **2.1^¶^** | **2.2^¶^** | **2.3^¶^** | **2.4** | **^¶^2.5^¶^** | **3.1^††^** | **3.2^††^** | **3.3^††^** | **3.4^††^** | **3.5^††^** | **4.1^‡‡^** | **4.2^‡‡^** | **4.3^‡‡^** | **4.4^‡‡^** | **4.5^‡‡^** | **5.1^§§^** | **5.2^§§^** | **5.3^§§^** | **5.4^§§^** | **5.5^§§^** |
| Banfield & Forbes (2018) | Yes | Yes | Yes | Yes | Yes | Yes | Yes |  |  |  |  |  |  |  |  |  |  | Yes | No | Yes | No | No | Yes | Yes | Yes | Yes | Yes |
| Bradley et al. (2021) | Yes | Yes | Yes | Yes | Yes | Yes | Yes |  |  |  |  |  |  |  |  |  |  |  |  |  |  |  |  |  |  |  |  |
| Carmel et al. (2017) | Yes | Yes | Yes | Yes | Yes | Yes | Yes |  |  |  |  |  |  |  |  |  |  |  |  |  |  |  |  |  |  |  |  |
| Coelho et al. (2024) | Yes | Yes | Yes | Yes | Yes | Yes | Yes |  |  |  |  |  |  |  |  |  |  |  |  |  |  |  |  |  |  |  |  |
| Donaghay-Spire et al. (2015) | Yes | Yes | Yes | Yes | Yes | Yes | Yes |  |  |  |  |  |  |  |  |  |  |  |  |  |  |  |  |  |  |  |  |
| Eiroa-Orosa et al. (2025) | Yes | Yes | Yes | Yes | Yes | Yes | Yes |  |  |  |  |  |  |  |  |  |  |  |  |  |  |  |  |  |  |  |  |
| Faith et al. (2023) | Yes | Yes | Yes | Yes | Yes | Yes | Yes |  |  |  |  |  |  |  |  |  |  |  |  |  |  |  |  |  |  |  |  |
| Forchuk et al. (2021) | Yes | Yes | Yes | Yes | Yes | Yes | Yes |  |  |  |  |  |  |  |  |  |  |  |  |  |  |  |  |  |  |  |  |
| Hammervold et al. (2022) | Yes | Yes | Yes | Yes | Yes | Yes | Yes |  |  |  |  |  |  |  |  |  |  |  |  |  |  |  |  |  |  |  |  |
| Horgan et al. (2021) | Yes | Yes | Yes | Yes | Yes | Yes | Yes |  |  |  |  |  |  |  |  |  |  |  |  |  |  |  |  |  |  |  |  |
| Howell et al. (2023) | Yes | Yes |  |  |  |  |  |  |  |  |  |  |  |  |  |  |  | Yes | No | Yes | No | Yes |  |  |  |  |  |
| Hristodoulidis et al. (2022) | Yes | Yes | Yes | Yes | Yes | Yes | Yes |  |  |  |  |  |  |  |  |  |  |  |  |  |  |  |  |  |  |  |  |
| Hyde et al. (2015) | Yes | Yes | Yes | Yes | Yes | Yes | Yes |  |  |  |  |  |  |  |  |  |  |  |  |  |  |  |  |  |  |  |  |
| Igarachi et al. (2024) | Yes | Yes | Yes | Yes | Yes | Yes | Yes |  |  |  |  |  |  |  |  |  |  |  |  |  |  |  |  |  |  |  |  |
| Isobel (2019) | Yes | Yes | Yes | Yes | Yes | Yes | Yes |  |  |  |  |  | Can´t  tell | Yes | Yes | No | Yes |  |  |  |  |  | Yes | Yes | Yes | Yes | Yes |
| Jørgensen et al. (2022) | Yes | Yes | Yes | Yes | Yes | Yes | Yes |  |  |  |  |  |  |  |  |  |  |  |  |  |  |  |  |  |  |  |  |
| Karbouniaris et al. (2022) | Yes | Yes | Yes | Yes | Yes | Yes | Yes |  |  |  |  |  |  |  |  |  |  |  |  |  |  |  |  |  |  |  |  |
| Keefe et al. (2020) | Yes | Yes | Yes | Yes | Yes | Yes | Yes |  |  |  |  |  |  |  |  |  |  |  |  |  |  |  |  |  |  |  |  |
| Kehoe et al. (2023) | Yes | Yes | Yes | Yes | Yes | Yes | Yes |  |  |  |  |  |  |  |  |  |  |  |  |  |  |  |  |  |  |  |  |
| Kidd et al. (2015) | Yes | Yes | Yes | Yes | Yes | Yes | Yes |  |  |  |  |  |  |  |  |  |  |  |  |  |  |  |  |  |  |  |  |
| Kirkegaard Thomsen et al. (2024) | Yes | Yes | Yes | Yes | Yes | Yes | Yes |  |  |  |  |  |  |  |  |  |  |  |  |  |  |  |  |  |  |  |  |
| Klevan et al. (2024) | Yes | Yes | Yes | Yes | Yes | Yes | Yes |  |  |  |  |  |  |  |  |  |  |  |  |  |  |  |  |  |  |  |  |
| Lauzier-Jobin & Houle (2024) | Yes | Yes | Yes | Yes | Yes | Yes | Yes |  |  |  |  |  |  |  |  |  |  |  |  |  |  |  |  |  |  |  |  |
| Molin et al. (2020) | Yes | Yes | Yes | Yes | Yes | Yes | Yes |  |  |  |  |  |  |  |  |  |  |  |  |  |  |  |  |  |  |  |  |
| Molin et al. (2018) | Yes | Yes | Yes | Yes | Yes | Yes | Yes |  |  |  |  |  | Can’t tell | Yes | Can’t tell | Can’t tell | Can’t tell |  |  |  |  |  | Yes | Yes | Yes | Yes | Yes |
| Okumura & Katsuki (2024) | Yes | Yes |  |  |  |  |  |  |  |  |  |  |  |  |  |  |  | Can’t tell | No | Yes | No | Yes |  |  |  |  |  |
| Pelto-Piri et al. (2019) | Yes | Yes | Yes | Yes | Yes | Yes | Yes |  |  |  |  |  |  |  |  |  |  |  |  |  |  |  |  |  |  |  |  |
| Pfeiffer et al. (2019) | Yes | Yes |  |  |  |  |  | Can’t Tell | Can’t Tell | No | No | Can’t Tell |  |  |  |  |  |  |  |  |  |  |  |  |  |  |  |
| Priebe et al. (2018) | Yes | Yes | Yes | Yes | Yes | Yes | Yes |  |  |  |  |  |  |  |  |  |  |  |  |  |  |  |  |  |  |  |  |
| Prytz et al. (2019) | Yes | Yes | Yes | Yes | Yes | Yes | Yes |  |  |  |  |  |  |  |  |  |  |  |  |  |  |  |  |  |  |  |  |
| Raitakari et al. (2018) | Yes | Yes | Yes | Yes | Yes | Yes | Yes |  |  |  |  |  |  |  |  |  |  |  |  |  |  |  |  |  |  |  |  |
| Reed et al. (2018) | Yes | Yes | Yes | Yes | Yes | Yes | Yes |  |  |  |  |  |  |  |  |  |  |  |  |  |  |  |  |  |  |  |  |
| Reinius et al. (2023) | Yes | Yes | No | No | Can’t tell | No | No |  |  |  |  |  |  |  |  |  |  |  |  |  |  |  |  |  |  |  |  |
| Rooney et al. (2016) | Yes | Yes | Yes | Yes | Yes | Yes | Yes |  |  |  |  |  |  |  |  |  |  |  |  |  |  |  |  |  |  |  |  |
| Scheirich et al. (2024) | Yes | Yes | Yes | Yes | Yes | Yes | Yes |  |  |  |  |  |  |  |  |  |  | Yes | Can’t tell | Yes | Yes | Yes | Yes | Yes | Yes | Yes | Yes |
| Sellin et al. (2018) | Yes | Yes | Yes | Yes | Yes | Yes | Yes |  |  |  |  |  |  |  |  |  |  |  |  |  |  |  |  |  |  |  |  |
| Sellin et al. (2019) | Yes | Yes | Yes | Yes | Yes | Yes | Yes |  |  |  |  |  |  |  |  |  |  | No | No | Yes | Yes | No | Yes | Yes | Yes | Yes | Yes |
| Selvin et al. (2021) | Yes | Yes | Yes | Yes | Yes | Yes | Yes |  |  |  |  |  |  |  |  |  |  |  |  |  |  |  |  |  |  |  |  |
| Shue et al. (2023) | Yes | Yes | Yes | Yes | Yes | Yes | Yes |  |  |  |  |  |  |  |  |  |  |  |  |  |  |  |  |  |  |  |  |
| Solomon et al. (2021) | Yes | Yes | Yes | Yes | Yes | Yes | Yes |  |  |  |  |  |  |  |  |  |  |  |  |  |  |  |  |  |  |  |  |
| Twamley et al. (2021) | Yes | Yes | Yes | Yes | Yes | Yes | Yes |  |  |  |  |  |  |  |  |  |  |  |  |  |  |  |  |  |  |  |  |
| Vandewalle et al. (2019) | Yes | Yes | Yes | Yes | Yes | Yes | Yes |  |  |  |  |  |  |  |  |  |  |  |  |  |  |  |  |  |  |  |  |
| van Lankeren et al. (2020) | Yes | Yes | Yes | Yes | Yes | Yes | Yes |  |  |  |  |  |  |  |  |  |  |  |  |  |  |  |  |  |  |  |  |
| Waldemar et al. (2019) | Yes | Yes | Yes | Yes | Yes | Yes | Yes |  |  |  |  |  |  |  |  |  |  |  |  |  |  |  |  |  |  |  |  |
| Walde et al. (2023) | Yes | Yes | Yes | Yes | Yes | Yes | Yes |  |  |  |  |  |  |  |  |  |  |  |  |  |  |  |  |  |  |  |  |
| Wallace et al. (2016) | Yes | Yes | Yes | Yes | Yes | Yes | Yes |  |  |  |  |  |  |  |  |  |  |  |  |  |  |  |  |  |  |  |  |
| Whittle et al. (2024) | Yes | Yes | Yes | Yes | Yes | Yes | Yes |  |  |  |  |  |  |  |  |  |  |  |  |  |  |  |  |  |  |  |  |
| Wong et al. (2019) | Yes | Yes |  |  |  |  |  |  |  |  |  |  |  |  |  |  |  | Yes | No | Yes | No | Yes |  |  |  |  |  |
| Zetterström et al. (2023) | Yes | Yes | Yes | Yes | Yes | Yes | Yes |  |  |  |  |  |  |  |  |  |  |  |  |  |  |  |  |  |  |  |  |
| *Note.* Table adapted from the MMAT (2018) used to evaluate methodological quality across five categories of empirical research (1 = *qualitative*, 2 = *quantitative randomised*, 3 = *quantitative non-randomised*, 4 = *quantitative descriptive*, 5 = *mixed methods*). **Screening questions (S.1, S.2):** S.1 = “Are there clear research questions?”; S.2 = “Do the collected data allow addressing the research questions?” **Qualitative criteria (1.1–1.5):** 1.1 = “Is the qualitative approach appropriate to answer the research questions”; 1.2 = “Are the qualitative data collection methods adequate to address the research questions?”; 1.3 = “Are the findings adequately derived from the data?”; 1.4 = “Is the interpretation of results sufficiently substantiated by data?”; 1.5 = “Is there coherence between qualitative data sources, collection, analysis, and interpretation?” **Quantitative randomised criteria (2.1–2.5):** 2.1 = “Is randomisation appropriately performed?”; 2.2 = “Are the groups comparable at baseline?”; 2.3 = “Are there complete outcome data?” 2.4 = “Are outcome assessors blinded to the intervention provided?”; 2.5 = “Did the participants adhere to the assigned intervention?” **Quantitative non-randomised criteria (3.1–3.5**): 3.1 = “Are the participants representative of the target population?”; 3.2 = “Are measurements appropriate regarding both the outcome and intervention (or exposure)?”; 3.3 = “Are there complete outcome data?”; 3.4 = “Are the confounders accounted for in the design and analysis?”; 3.5 = “During the study period, was the intervention administered (or exposure occurred) as intended?” **Quantitative descriptive criteria (4.1–4.5):** 4.1 = “Is the sampling strategy relevant to address the research questions? “; 4.2 = “Is the sample representative of the target population?”; 4.3 = “Are the measurements appropriate??”4.4 = “Is the risk of nonresponse bias low?”; 4.5 = “Is the statistical analysis appropriate to answer the research questions?” **Mixed-methods criteria (5.1–5.5):** 5.1 = “Is there an adequate rationale for using a mixed-methods design to address the research questions? “; 5.2 = “Are the different components of the study effectively integrated to answer the research questions?”; 5.3 = “Are the outputs of the integration of qualitative and quantitative components adequately interpreted?”; 5.4 = “Are divergences and inconsistencies between quantitative and qualitative results adequately addressed? “; 5.5 = “Do the different components of the study adhere to the quality criteria of each tradition of the methods involved?” **Response key:** “Yes” = Criterion clearly met; “No” = Criterion clearly not met; “Can’t tell” = Insufficient or unclear information to judge. | | | | | | | | | | | | | | | | | | | | | | | | | | | |
